# AquIRE reveals the mechanisms of clinically induced RNA damage and the conservation and dynamics of glycoRNAs

**DOI:** 10.1093/nar/gkag080

**Published:** 2026-02-05

**Authors:** Zijian Zhang, Zornitsa Vasileva Kotopanova, Kexin Dang, Xiangxu Kong, Nicole Simms, Tin Wai Yuen, Lan Lam, Lauren Forbes Beadle, Emma Hilton, Taqdees Qureshi, Marianna Coppola, Callum David Holmes, Kwan Ting Kan, Mark Ashe, Patrick Gallois, Hilary Ashe, Michael Braun, Mark Saunders, Paul Sutton, David J Thornton, John R P Knight

**Affiliations:** Division of Cancer Sciences, Faculty of Biology, Medicine and Health, The University of Manchester, Manchester M13 9NT, United Kingdom; Division of Cancer Sciences, Faculty of Biology, Medicine and Health, The University of Manchester, Manchester M13 9NT, United Kingdom; Division of Cancer Sciences, Faculty of Biology, Medicine and Health, The University of Manchester, Manchester M13 9NT, United Kingdom; Division of Cancer Sciences, Faculty of Biology, Medicine and Health, The University of Manchester, Manchester M13 9NT, United Kingdom; Division of Cancer Sciences, Faculty of Biology, Medicine and Health, The University of Manchester, Manchester M13 9NT, United Kingdom; Division of Cancer Sciences, Faculty of Biology, Medicine and Health, The University of Manchester, Manchester M13 9NT, United Kingdom; Division of Cancer Sciences, Faculty of Biology, Medicine and Health, The University of Manchester, Manchester M13 9NT, United Kingdom; Faculty of Biology, Medicine and Health, University of Manchester, Manchester M13 9PT, United Kingdom; Manchester Cell Matrix Centre and Lydia Becker Institute of Immunology and Inflammation, School of Biological Sciences, Faculty of Biology, Medicine and Health, Manchester Academic Health Sciences Centre, The University of Manchester, Manchester M13 9PT, United Kingdom; Colorectal and Peritoneal Oncology Centre, The Christie NHS Foundation Trust, Manchester M20 4BX, United Kingdom; Faculty of Biology, Medicine and Health, University of Manchester, Manchester M13 9PT, United Kingdom; Faculty of Biology, Medicine and Health, University of Manchester, Manchester M13 9PT, United Kingdom; Faculty of Biology, Medicine and Health, University of Manchester, Manchester M13 9PT, United Kingdom; Faculty of Biology, Medicine and Health, University of Manchester, Manchester M13 9PT, United Kingdom; Faculty of Biology, Medicine and Health, University of Manchester, Manchester M13 9PT, United Kingdom; Faculty of Biology, Medicine and Health, University of Manchester, Manchester M13 9PT, United Kingdom; Division of Cancer Sciences, Faculty of Biology, Medicine and Health, The University of Manchester, Manchester M13 9NT, United Kingdom; Colorectal and Peritoneal Oncology Centre, The Christie NHS Foundation Trust, Manchester M20 4BX, United Kingdom; Division of Cancer Sciences, Faculty of Biology, Medicine and Health, The University of Manchester, Manchester M13 9NT, United Kingdom; Colorectal and Peritoneal Oncology Centre, The Christie NHS Foundation Trust, Manchester M20 4BX, United Kingdom; Division of Cancer Sciences, Faculty of Biology, Medicine and Health, The University of Manchester, Manchester M13 9NT, United Kingdom; Colorectal and Peritoneal Oncology Centre, The Christie NHS Foundation Trust, Manchester M20 4BX, United Kingdom; Manchester Cell Matrix Centre and Lydia Becker Institute of Immunology and Inflammation, School of Biological Sciences, Faculty of Biology, Medicine and Health, Manchester Academic Health Sciences Centre, The University of Manchester, Manchester M13 9PT, United Kingdom; Division of Cancer Sciences, Faculty of Biology, Medicine and Health, The University of Manchester, Manchester M13 9NT, United Kingdom

## Abstract

RNA is subject to many modifications, from small chemical changes like methylation to conjugation of biomolecules such as glycans. As well as endogenously written modifications, RNA is also exposed to damage induced by its environment. Certain clinical compounds are known to covalently modify RNA with a growing appreciation of how these impact clinical efficacy. To understand the regulation of these modifications, we need a reliable, sensitive, and rapid methodology for their quantification. Thus, we developed Aqueous Identification of RNA Elements (AquIRE) and applied it to the analysis of drug-induced RNA damage by 5FU, oxaliplatin, and temozolomide in clinically relevant cell models. We demonstrate that RNA damage is widespread and follows previously unappreciated temporal dynamics. AquIRE also provides a highly sensitive method to detect RNAs modified by glycans. We leverage this to expand the horizons of the glycoRNA world across the kingdoms of life as well as identifying cell-free glycoRNAs in multiple species. We demonstrate that glycoRNA expression is dynamic during embryo development, modulated during senescence, and elevated by RNA-damaging agents. Finally, we use RNA digestion to demonstrate that cell surface or cell-free RNA promotes the cytotoxicity of RNA-damaging chemotherapy. Together, the AquIRE platform provides an intrinsically flexible method to study diverse RNA modifications from any sample.

## Graphical Abstract

**Graphical Abstract:**
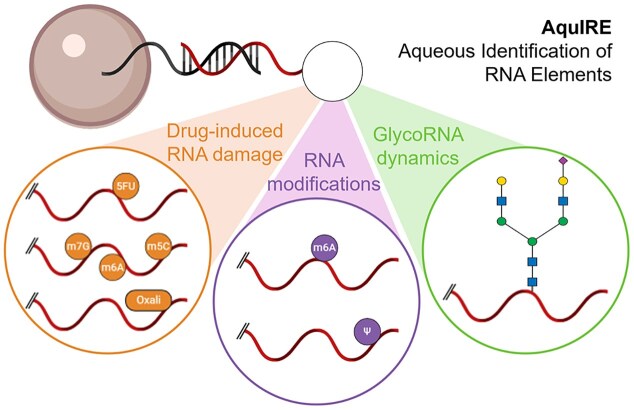

## Introduction

Antimetabolites or alkylating agents are administered to over 2 million people worldwide each year to manage diseases including cancer, autoimmunity, and viral infections [1, 2]. These agents are multifaceted in their mechanisms of action, leading to detrimental side effects along with clinical benefits. The advent of targeted therapies has meant a sharp decline in approvals for new antimetabolite or alkylating agents, yet for many diseases, they remain the best or only clinical options. In fact, globally the usage of these drugs continues to grow. Mechanistically, these compounds cause damage to multiple target biomolecules, which occurs in a specific manner related to the chemical structure of the drug. Surprisingly, given their extensive clinical use, their exact mechanisms of action remain poorly understood.

Our knowledge of chemotherapies has traditionally been DNA-centric with their effects on other biomolecules considered unimportant compared to DNA damage. Recent reports have challenged this dogma and presented RNA as an important target for clinical compounds [1, 3, 4]. For instance, it is known that incorporation of the antimetabolite 5FU into RNA correlates with its cytotoxicity [5–8], while the pseudo-alkylating agent oxaliplatin impacts RNA-related functions, unlike its related compounds cisplatin and carboplatin [9]. Highlighting the biological importance of RNA damage, the cell’s intricate molecular mechanisms to detect and manage its impact have recently been described [10–16]. This work has primarily used tool compounds and physical stresses such as irradiation, raising the question of when these pathways become engaged in the physiological setting. Addressing this, roles for RNA damage pathways have recently been shown to impact inflammation, stem cell function, and aging [17–20]. With some RNAs known to be as long lived as DNA in non-mitotic cells [21], understanding the physiological causes and dynamics of RNA damage is a pressing need.

To understand RNA as a direct drug target, the field requires an easy-to-implement method to directly measure specific RNA damage from multiple compounds, ideally from low-input samples. With this in mind, we developed AquIRE—Aqueous Identification of RNA Elements—and used this to quantify relative levels of direct RNA damage from the antimetabolite 5FU and the (pseudo)alkylating agents oxaliplatin and temozolomide. Our methodology is sensitive and has intrinsic flexibility for the desired target of interest. As such, we further benchmarked AquIRE for the analysis of two endogenously written chemical modifications, m6A and pseudouridine.

Finally, we leveraged the flexibility of the AquIRE platform to study glycoRNAs. GlycoRNAs are RNAs that are modified by glycans, broadly orthologous to protein or lipid glycosylation. Their expression has been detailed in mammalian model systems, as well as in liquid samples from patients [22–25]. However, these experiments require either the use of bio-orthogonal agents to label nascent glycoRNAs or complex imaging methodologies that are inaccessible to the wider research community and not amenable for high throughput analyses. Using AquIRE to detect glycoRNAs allows their analysis from as little as 10 ng of RNA using an easily accessible and highly reproducible protocol. We expand the known breadth of glycoRNA expression to include organisms from *Xenopus* to plants to single-celled microbes and prokaryotes. In parallel, we reveal glycoRNA expression dynamics during the earliest stages of development and in senescence. We also observed that glycoRNAs are present in liquid, cell-free, samples from seven different organisms, including the cell-free material from colorectal cancer cell line models, as well as primary resected tumours. Finally, we show that multiple RNA-damaging clinical compounds elevate glycoRNA expression, and that glycoRNAs on the cell surface or within cell-free media are required for the optimal cytotoxicity from 5FU.

Altogether, this work provides a new method to identify and study RNA elements that will address key questions for RNA biologists. We apply our methodology to reveal the pervasive effects of drug-induced RNA damage on RNA biology, showing the relationship between widely used drugs, the epitranscriptome and glycoRNA expression.

## Materials and methods

All materials used in the study are listed in Supplementary Table S1.

### *In vivo* animal studies

*Mus musculus*: Adult wild-type mixed background animals of both genders were used in this study. Animals were kept under Establishment Licence number X44772EDA, granted by the UK Home Office, in individually ventilated cages with *ad libidum* access to water and diet in a 12:12 light cycle. *Xenopus tropicalis*: Adult male and female frogs were primed with 15 units of pregnant mare serum gonadotrophin (MSD Animal Health) 18–24 h prior to ovulation. Mating was subsequently induced with 50 units of human chorionic gonadotrophin (MSD Animal Health) in males and 75 units in females. Hormone injection in adults was performed under United Kingdom Home Office animal project licence number PFDA14F2D. All data presented in this study were obtained from pre-feeding stage embryos (~3–4 days of development from fertilization), which are not considered protected animals for regulated procedures under the Animals (Scientific Procedures) Act 1986. All experiments using *Xenopus tropicalis* animals are reported according to applicable ARRIVE guidelines for this species. For the ionomycin secretagogue protocol, experiments were performed as previously published [26]. Batches of 50–60 *Xenopus tropicalis* embryos at NF stage 40 were transferred into 3 ml 0.01 × MMR media [0.1 M NaCl, 2 mM KCl, 1 mM MgSO_4_, 2 mM CaCl_2_, 5 mM HEPES, pH 7.8, 0.1 mM ethylenediaminetetraacetic acid (EDTA)] in single wells of a 12-well plate and incubated at 25°C. Embryos were exposed to 4 µM ionomycin (Sigma–Aldrich) and incubated at room temperature for 10 min with gentle swirling each minute. Media were centrifuged at 10 000 × *g* for 5 min to pellet cellular debris, and RNA extracted as outlined below for large-volume liquid samples. *Drosophila melanogaster: y*^1^*w*^67c23^ flies were housed in standard conditions. For ovary tissue samples, 8–10 ovary pairs from 2- to 5-day old non-virgin females were used. Embryos were harvested at the specified time points after being laid on apple juice agar plates supplemented with yeast paste in small cages.

### Human participants and tumour processing

Access to colorectal primary or metastatic tumours was granted by the Manchester Cancer Research Centre Biobank, application number 23_JOKN_01. Approval is under the MCRC Biobank Research Tissue Bank Ethics, reference 22/NW/0237. Anonymized details of the patients are available in Supplementary Table S2. 1000–4000 mg of tumour was place in ADF media and processed within 16 h of surgery. Tumours were cut into small pieces using scissors and/or scalpels then placed in gentleMACS C tubes (Militenyi) in at least 5 ml of ADF supplemented with 10 mM EDTA and 1.5 mg/ml collagenase II, and run on the 37C_h_TDK_1 gentleMACS program. After digestion, 150 µM collagenase inhibitor was added and the sample passed through a 100 µm cell strainer (Starlab), which was washed with 0.5 volume of ADF. Strained cells were then pelleted at 600 × *g* and washed twice in 5 ml of ADF. All three supernatants from these centrifugations were pooled and centrifuged at 1000 × *g* for 5 min. RNA from this cell-free media was then processed as detailed below for large-volume RNA samples. A fraction of the cell pellet was directly lysed in Zymo RNA lysis buffer.

### Plant models

Seedling growth conditions: *Arabidopsis thaliana* Col-0 seeds were sterilized for 10 min under rotation with a sterilizing solution (50% ethanol and 0.5% Triton X100), followed by five subsequent washes using sterile distilled water. Sterile seeds were sown onto sterile plates containing 1% glucose, 0.8% agar, and ½ Murashige and Skoog basal medium (Duchefa) and stratified for 2 days at 4°C in darkness. Seeds were grown in continuous light (69 μmol/m^2^s) at 24°C in a growth cabinet (Perceval, Perry, IA, USA) for 7 days. *Plant growth conditions*: Seeds were planted in Levington Advance F2, grown in 8 cm pots, and watered regularly. They were grown in controlled growth cabinets under a short-day photoperiod of 8 h light (112 μmol/m^2^s intensity) at 22°C during the day and 17°C during the night. To induce senescence, individual 5-week-old leaves were wrapped in aluminium foil without detaching the leaves from the plants. After 4 and 6 days, the dark-induced leaves were unwrapped, photographed, and total RNA extracted. The control leaves, unwrapped, were collected at day 6.

### Cell culture

All cell lines were maintained under standardized conditions at 37°C in a humidified atmosphere containing 5% CO_2_. Media were refreshed every 3–4 days, and cells were passaged using trypsin-EDTA when they reached ~80% confluence. Specific medium compositions for each cell line are detailed: LS174T, RKO, and DLD1 cells were grown in Minimum Essential Medium (MEM) supplemented with 10% fetal bovine serum (FBS), 2 mM L-glutamine, 1× NEAA, and 1% PenStrep. A172 and U251 were grown in Dulbecco’s modified Eagle’s medium (DMEM) supplemented with 10% FBS, 2 mM L-glutamine, and 1% PenStrep. HCT116 were grown in either the MEM or DMEM base media listed earlier. JVE-127 were cultured in RPMI supplemented with 10% FBS, 2 mM L-glutamine, and 1% PenStrep. JVE-253 were grown in RPMI supplemented with 10% FBS, 2 mM GlutaMAX, and 1% PenStrep.

### Microorganisms culture

*Saccharomyces cerevisiae* W303-1A were grown in SCD media (1× Yeast Nitrogen Base w/o amino acid, 1× Kaiser Complete SC media, and 2% D-glucose) and harvested at an OD_600_ of 0.8. One Shot TOP10 chemically competent *Escherichia coli* were cultured in Luria broth and harvested in exponential growth phase.

### Drug treatments

5FU, STM2457, temozolomide, ionomycin, NGI-1, and Benzyl-α-GalNAc were dissolved in DMSO, stored at −20°C, and used with 6 months or after five freeze-thaw cycles, whichever was sooner. Oxaliplatin and carboplatin were dissolved in water and kept at 4°C for a maximum of 1 week. Cisplatin was dissolved directly in cell culture media and kept at 4°C for a maximum of 1 week. All vehicle treatments used the same volume of drug-free DMSO/water/media.

### RNA isolations

RNA was isolated by Zymo’s Quick-RNA^TM^ Miniprep Kit with DNase digestion, unless otherwise stated, as per the manufacturer’s protocol recommendations. The lysis step differed across sample types. Human cell pellets were lysed by immediate incubation with Zymo RNA lysis buffer. Bacterial cell pellets, obtained by a 15-min-long centrifugation at 3000 × *g* at 4°C, were resuspended in phosphate buffered saline (PBS) and then lysed in three volumes of Zymo RNA lysis buffer. Mouse tissues were sampled into RNAlater (Invitrogen) and stored at −80°C prior to extraction. These were lysed in Zymo RNA lysis buffer using gentleMACS M tubes (Militenyi). Whole mouse blood from cardiac puncture was placed into heparin containing tubes (Teklab) and separated into blood cells and plasma by centrifugation at 10 000 × *g* for 5 min. Blood cells were directly lysed in Zymo RNA lysis buffer, while plasma was mixed with the same buffer at a ratio of one part plasma to three parts lysis buffer. Five whole *Xenopus* embryos were pooled and lysed directly in Zymo RNA lysis buffer with repeated pipetting. For large-volume liquid samples (cell line media, bacterial broth, cell-free human tumour material, polysome profile fractions, *Xenopus* MMR media, commercially available sera), these were mixed with an equal volume of 7.7 M guanidine hydrochloride, then an equal volume again of 100% ethanol, giving a final ratio of 1:1:2. This was stored at −20°C for at least 24 h then RNA pelleted at 4000 × *g* for 45 min. RNA pellets were dissolved in Zymo RNA lysis buffer. For *Drosophila* RNA extractions, embryos were dechorionated for 2 min in 50% bleach (2.5% final concentration of sodium hypochlorite diluted in distilled water) and rinsed thoroughly in distilled water. For ovaries, these were dissected in 1× PBS on ice, and transferred to 1.5 ml microcentrifuge tubes, and the PBS removed. Fifty microliters Trizol Reagent (Invitrogen) was added to the embryos or ovaries, and samples were crushed and homogenized using a disposable pestle (Fisher Scientific). An additional 450 μl Trizol was added to rinse the pestle, and then RNA was extracted and purified according to the manufacturer’s protocol. For *S. cerevisiae* RNA extractions, cells were pelleted at 1500 × *g* for 15 min at 4°C and immediately lysed in Trizol. Supernatant was centrifuged again at 15000 × *g* for 30 min at 4°C to yield cell debris, which was placed in Trizol. The supernatant was then concentrated ∼20-fold using a Vivaspin 20 column with 5 kDa cutoff (Sartorius), and RNA was extracted using Trizol. For *Arabidopsis* RNA extraction, the Aurum Total RNA Mini Kit from BioRad was used, following the manufacturer’s guidelines. RNA was extracted from 10 pooled seedlings or one leaf (up to 100 mg).

### *In vitro* transcription

All reactions used the HighYield T7 RNA Synthesis Kit from Jena Bioscience, following the manufacturer’s instructions. For incorporation of non-canonical nucleotides, these were substituted for the canonical nucleotide at the stated ratios. Specifically, 5FUTP or pseudoUTP were substituted for UTP or m6ATP for ATP. To template transcription, we used either the DNA provided with the HighYield kit or a pUC57-Curlcake 3 DNA, expressed and digested as previously published [27]. Reactions were incubated for 1.5 h at 37°C in a thermal cycler. The reaction volume was then adjusted to 50 µl, and RNA purified using the Monarch Spin RNA cleanup kit, and the RNA product stored at −80°C.

### RNA analysis and digestions

*RNA integrity number (RIN) analysis*: A 2100 Bioanalyzer was used with RNA 6000 Pico Kit set to Eukaryote Total RNA. RIN values were calculated using the Bioanalyzer software. RNA and DNA concentrations were routinely determined using a NanoDrop. *PNGase F digests*: 1.5 µg RNA was incubated with recombinant PNGase F (NEB) using 0.75 µl enzyme in a 10 µl reaction for 2 h at 37°C. A no-enzyme control was incubated in parallel. Subsequently, RNA was purified using RNA Clean and Concentrator columns (Zymo) before quantification and analysis of equal amounts of RNA by AquIRE. *RNase A digests*: Cells were treated with non-cell permeable RNase A (NEB) concurrently with drug treatments by diluting the enzyme directly in cell media.

### Aqueous Identification of RNA Elements

The input material for all AquIRE assays was purified RNA. Equal amounts of RNA per sample were used within each experiment. The RNA was polyadenylated using *E.Coli* polyA polymerase (NEB), unless otherwise stated, as per the manufacturer’s protocol recommendations. Incubation of the polyadenylated RNA in denaturing buffer (20 mM TRIS, pH 7.5, 200 mM NaCl, and 2% w/v sodium dodecyl sulphate) for 5 min at 65°C ensured removal of RNA secondary structures, following which the samples were rapidly cooled to 4°C. Magnetic oligodT beads (Cytiva) were washed twice with denaturing buffer before binding the RNA to the beads at a ratio of one µg of RNA per 10 µl of washed beads. A 5-min-long incubation at room temperature allowed oligodT:poly-A-tail base-pairing, which was followed by two washes, one with denaturing buffer and a subsequent one with wash buffer (20 mM TRIS, pH 7.5, 200 mM NaCl, and 1 mM EDTA). *Immunodetection:* Primary antibodies were applied at between 1:200 and 1:500 dilution, then recognized by species-specific biotinylated secondary antibodies. Secondary antibodies were subsequently bound by fluorescently labelled streptavidin (Invitrogen). Wash buffer was used as the diluent for all antibodies and streptavidin. Degradation of RNA was prevented by the addition of RiboLock (Thermo Scientific), at 0.1 U/μl. All incubation steps were performed on a shaker for an hour at room temperature except the primary antibody incubation, which lasted for an hour and a half. Each incubation step was followed by two wash steps using wash buffer removing any unbound material. *Glycan detection*: All glycan detection steps were the same as the AquIRE steps described above, apart from the immunodetection step, replaced by glycan-binding biotinylated lectins. A dilution factor of 1:200 was used for the preparation of the biotinylated lectin incubation solution. The lectin incubation was performed on a shaker for 1.5 h at room temperature. *Aqueous elution and signal detection*: The complexes of RNAs and antibodies/lectins of interest were eluted using nuclease-free water, which was incubated for 10 min at room temperature. Fluorescence reading in black opaque 96-well plates was conducted using a microplate reader and SkanIt™ Software (Thermo Scientific). The specified excitation and emission wavelengths were 488 and 515 nm, respectively. *Data analysis*: The raw fluorescence values presented are an average of triplicate reads from the final eluate of each sample after removal of outliers. Where the AquIRE signal has been normalized, the signal of the negative control is subtracted then values presented relative to a given sample, either set to 1 or 0. For glycoRNA samples the fluorescence is additionally normalized against *in vitro* transcribed (IVT) RNA (i.e. with no glycoRNAs). Where the fluorescence is presented per µg of RNA, the mean fluorescence reads are divided by the amount of input RNA. The distribution of RNA and glycoRNA signal across different fractions, such as cellular and cell-free, was calculated as follows. The total RNA content of each fraction was the concentration of the sample, determined by Nanodrop, multiplied by the volume. This gives a yield of RNA, which is summed for all fractions then presented as a percent of that total for each fraction. Similarly, the glycoRNA content within a known quantity RNA was determined by AquIRE for each fraction, giving a value for the fluorescence per µg of RNA. This was then multiplied by the total RNA quantity in each fraction and summed to give the total glycoRNA expression. Again, percentages of this total are presented for each fraction.

### RNA dot blot

Nitrocellulose membranes (Fisher Scientific) were soaked in sterile distilled water, washed in 10× SSC (1.5M NaCl, 150 mM tri-sodium citrate), and air-dried. RNA samples were thawed on ice, mixed with three volumes of RNA incubation solution (66% formamide, 8.5% formaldehyde, 150 mM MOPS, 70 mM sodium acetate, 7.7 mM EDTA, pH 7), heated at 65°C for 5 min, and cooled on ice. An equal volume of ice-cold 10× SSC was added. The RNA solution was dotted onto the membrane and air dried. Membranes were exposed to 130 kJ of UV_254 nM_ in a crosslinker. Total RNA was visualized with methylene blue for 5 min, washed with water until the membrane turned white again, and air-dried. For immunodetection, the membranes were blocked with 5% non-fat dry milk in TBST for 1 h, then incubated overnight at 4°C with primary antibodies. After washing, the membranes were incubated with HRP-conjugated secondary antibody for 1 h. Protein bands were detected using Clarity™ Western ECL substrate (Fisher Scientific) and visualized. Dot intensity was determined using ImageJ.

### Nucleotide modification quantification by LC-MS

Samples were analysed by Arraystar Inc (Rockville, MD) following quality control assessment. We present quantification of a subset of modifications from the full panel assessed by Arraystar, focusing on methylation. The raw data from this LC-MS experiment can be found in Supplementary Table S3.

### Immunofluorescence and analysis

Staining and single-cell analysis of 5FU incorporation into RNA was performed as previously described [28]. Images were taken using confocal microscopy (Olympus Spinning Disk) at 40× magnification.

### Complementary DNA stalling assays

SuperScript™ II Reverse Transcriptase kit (Invitrogen) was used with random primers (Promega), dNTP Mix (Invitrogen), and RiboLock (Thermo) to generate complementary DNAs (cDNAs) from total RNA in a total reaction volume of 20 μl, as per the kit recommendations. The amount of random primers used for an RNA input amount of 1000 ng was 200 ng, and it was decreased or increased proportionally if the RNA input was smaller or greater, respectively. The generated cDNAs were then used in a 10 μl qPCR, including PowerTrack™ SYBR™ Green Master Mix (Applied Biosystems) and target-specific primers (Integrated DNA Technologies), as per the kit recommendations. qPCRs were run on the QuantStudio 5-C Real-Time PCR System within 384-well format plates covered with adhesive seal using Thermo Scientific™ Design and Analysis Software 2. The run method involved a 2-min long hold step at 21°C and a subsequent 2-min-long Taq DNA polymerase-activating step at 95°C, which was followed by 40 thermal cycles of 15-s-long denaturation at 95°C and a minute-long primer annealing and extension at 60°C. Normalization was done against the transcripts from the housekeeping genes actin beta (*ACTB*) and glyceraldehyde-3-phosphate dehydrogenase (*GAPDH*), which was followed by normalization against the control groups. Normalization was done against *ACTB* and *GAPDH*, as their raw Ct values were not changed, which was speculated to be due to their low abundance compared to the RNAs of interest. The ΔΔCt method was used with the Ct of measured transcript normalized against the average of the Cts for *ACTB* and *GAPDH* for all samples. The difference between this ΔCt for experimental group and the control group then yielded a ΔΔCt value. Control samples are set to 1, and values for experimental conditions are a fold change to this. Interpretation of results was based on the inverse proportionality between RT-qPCR signal and drug-induced RNA damage.

### Polysome profiling

This was performed as previously [29]. Briefly, exponentially growing cells were treated with 200 µg/ml cycloheximide for 3 min, then transferred to ice. Media was removed and replaced with ice-cold PBS also containing cycloheximide for detachment by scraping. Pelleted and washed cells were lysed (300 mM NaCl, 15 mM MgCl_2_, 15 mM TRIS, pH7.5, 100 µg/ml cycloheximide, 0.1% Triton X-100, 2 mM DTT, and 0.2 U/ml RiboLock). Post-nuclear supernatants were then layered on 10%–50% sucrose gradients containing the same concentration of NaCl, MgCl_2_, Tris, and cycloheximide as in the lysis buffer. These were then centrifuged in a JXN-30 centrifuge with a JS24.15 rotor at 79 000 × *g* at 4°C for 3.5 h. Gradients were separated through a live UV_254 nm_ detector and distributed into nine 1.4 ml fractions. For RNA isolation, sub-polysome and polysome fractions were pooled, and RNA was extracted from the resulting large volume liquid samples as described earlier.

### Statistical analysis and software

All statistical analyses were performed using GraphPad Prism 10, with details of the individual tests used found in the figure legends. Data are presented as the mean, and error bars are the standard error of the mean (SEM), unless otherwise stated. The number and type of replicates are also depicted in each figure legend. *P* values of 0.05 or less were considered significant. Glycans were drawn using GlycoGlyph [30].

## Results

### AquIRE detects 5FU:RNA localization and dynamics

We have previously used an anti-BrdU antibody to detect 5FU in RNA at single cell resolution [28]. Here, we used this same antibody in an RNA dot blot to quantify 5FU incorporation into RNA after treatment of colorectal cancer cells (HCT116). In parallel, we analysed IVT RNA made in the presence of 5FUTP, as a positive control. Unexpectedly, we observed no signal from cellular RNA (Supplementary Fig. S1A) under the same conditions where we have previously observed 5FU in RNA by immunofluorescence [28]. We were able to detect a signal from IVT RNA with 100% 5FUTP incorporation, but this signal dropped off sharply at 75% and was barely visible at 25%, resulting in a non-significant correlation between 5FU content and signal (Supplementary Fig. S1B). We hypothesized that immobilization of purified RNA on membranes occludes or destroys the epitope. Consistently, the fluorine epitope of 5FU sits on the Hoogsteen base edge, while membranes are designed to display the Watson–Crick edge for hybridization [1]. Furthermore, adducts of 5FU that occur when crosslinking RNA to membranes result from defluorination [31], which would destroy the epitope we want to measure.

**Figure 1.**
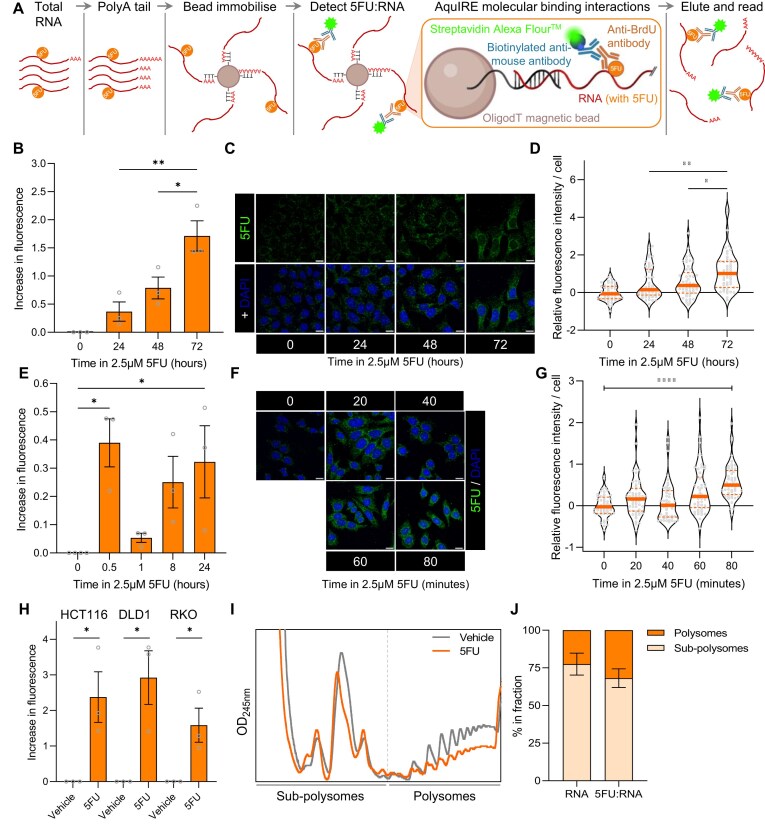
Detecting RNA damage by 5FU using AquIRE. (**A**) Schematic of the experimental protocol for AquIRE. Total RNA, containing modifications (e.g. 5FU), is polyA-tailed, then immobilized on oligodT beads. RNA is sequentially exposed to primary antibody to detect RNA elements, a biotin-tagged secondary antibody then Alexa Fluor^TM^-tagged streptavidin. Finally, water is used to elute a fluorescent signal. Figure in part generated in BioRender: https://BioRender.com/f11o153. (**B**) HCT116 cells were treated with 2.5 µM 5FU for the indicated times, RNA extracted, then equal amounts of RNA analysed for RNA content. The graph shows AquIRE fluorescent measurements normalized against vehicle treatment set to 0. Bars represent the average of three biological replicates, each shown as grey circles and the error bars are SEM. Significance was calculated by analysis of variance (ANOVA) using Šídák multiple comparison testing. (**C**) HCT116 cells were treated as in panel (A) then stained for 5FU incorporation into RNA and counterstained with DAPI. Scale bar 50 µm. (**D**) Violin plots of the quantification of the 5FU incorporation in cells as shown in panel (C). Each grey circle represents one of 50 individual cells analysed per timepoint in this biological replicate. The thick orange lines are the mean and dashed lines are quartiles. Data are presented relative to vehicle treatment (0 h), which is set to 0. The black line extending from the *y-*axis shows the average of vehicle treatment. Significance was determined by Kruskal–Wallis analysis with Dunn’s multiple-comparison testing. Significant differences between drug-treated groups are annotated. All treatments were significantly different to 0 h. (**E**) HCT116 cells were treated with 5FU at 2.5 µM for the times shown, and analysis performed as in panel (A). Bars represent the average of at least three biological replicates for each timepoint, shown as grey circles, and the error bars are SEM. Significance was analysed by ANOVA. (**F**) HCT116 were treated with 2.5 µM 5FU for the indicated times and presented as in panel (C). Scale bar 50 µm. (G) 5FU intensity per cell from panel (F) was calculated for 50 cells per indicated timepoint. Data are presented in violin plots as described in panel (D). Significance was calculated using a Kruskal–Wallis test without multiple/individual comparisons. (**H**) RNA was analysed from HCT116, DLD1, or RKO cells were treated with vehicle or 10 µM 5FU for 72 h. Graph shows the levels of 5FU incorporation relative to the vehicle set to 0. Data are *n* = 3 biological replicates, with error bars showing SEM. Significance was determined by unpaired *t*-test. (**I**) HCT116 cells were treated with 10 µM 5FU for 24 h then their cytoplasmic fraction separated by sucrose density gradient. From these gradients, OD_254 nm_ polysome traces were obtained and overlaid here. Data are representative of two biological replicates. (**J**) RNA was extracted from the sub-polysome and polysome fractions of the 5FU treated sample shown in panel (I), with total RNA distribution between the fractions (left) and 5FU:RNA content determined by AquIRE (right) plotted ± SEM. *P < .05, **P < .01, ****P < .0001.

Thus, we sought to develop an alternative, membrane-independent approach to biochemically detect 5FU incorporation in purified RNA. We opted for aqueous detection to maximize the surface area of RNA and number of epitopes available. We took advantage of the interaction between RNA polyA tails and commercially available oligodT beads to tether RNA through their 3′ ends, while leaving the full surface area of the remaining sequence accessible in aqueous solution. Crucially, we developed this method using buffers amenable to coincubation with protein-based detection reagents and coupled this with the enzymatic addition of polyA tails to total RNA using commercially available polyA polymerase. We termed this method AquIRE for Aqueous Identification of RNA Elements (Fig. 1A).

First, we used IVT 5FU-containing RNAs as detailed above and saw a strong correlation between 5FU content and fluorescent signal (Supplementary Fig. S1C). Next, we asked if AquIRE could detect 5FU incorporation into total RNA extracted from HCT116 colorectal cancer cells treated with clinically achievable doses of 5FU over a 72-h time course. This revealed a steady increase in 5FU in RNA with increased exposure time (Fig. 1B), correlating perfectly with 5FU incorporation measured using our previously published immunofluorescent technique (Fig. 1C and D; Supplementary Fig. S1D). We then analysed shorter, more clinically relevant timepoints. The method showed sensitivity, being able to detect 5FU incorporation using as little as 100 ng of RNA. Importantly, we were able to recover >100% of the RNA input at the end of the experiment, showing that the sample RNA is well retained on the oligodT beads, while the increase in RNA recovery compared to input is likely due to the addition of poly(A) tails (Supplementary Fig. S1E).

The short time course revealed previously unknown dynamics in the incorporation of 5FU into RNA. We observed a consistent peak at 30 min, followed by a marked reduction at 1 h, preceding a steady increase back to levels seen at 30 min by 24 h (Fig. 1E). Again, this result was recapitulated by immunofluorescence, identifying a biphasic pattern in 5FU incorporation into RNA (Fig. 1F and G; Supplementary Fig. S1F). Expanding this, we confirmed the incorporation of 5FU into RNA in multiple colorectal cancer cell lines (Fig. 1H) and that 5FU is present in translating polysomes (Fig. 1I and J). 5FU treatment reduced polysome abundance but RNA containing 5FU is not occluded from polysomes. Both observations are consistent with a previous report [8] as well as our previous work that 5FU induces a robust ribosome quality control response [28]. Together this indicates that 5FU:RNA participates in translation.

### AquIRE identifies divergent trophism for RNA between different platinum agents

**Figure 2.**
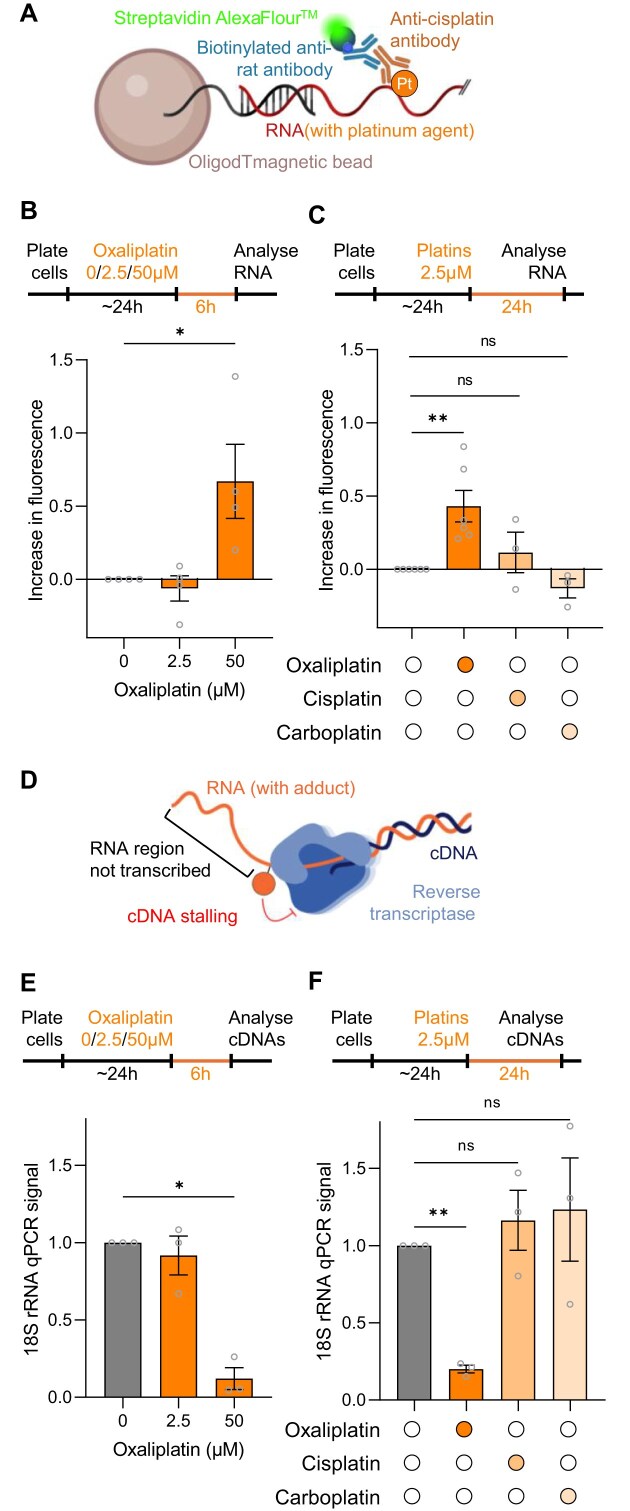
Oxaliplatin, but not other platins, causes detectable RNA damage. (**A**) Schematic of oxaliplatin:RNA detection by AquIRE. (**B**) Top, timeline of drug treatments outlining that HCT116 cells were treated with increasing doses of oxaliplatin for 6 h prior to extraction and analysis of total RNA. Bottom, graph plotting the average of four biological replicates detecting oxaliplatin by AquIRE in RNA after 0, 2.5, and 50 µM treatments. Bar represents the mean ± SEM, normalized against 0 µM which is set to 0. Significance was calculated by ANOVA using Šídák multiple comparison testing. (**C**) Top, timeline of drug treatments of HCT116 cells, which were treated with 2.5 µM of each platinum agent for 24 h prior to analysis of RNA. Bottom, bar graph shows the levels of platinum agent detected in RNA, shown as the average of ≥3 biological replicates. Statistical significance, or lack thereof, was determined by ANOVA using Šídák multiple comparison testing. (**D**) Schematic of the cDNA reverse transcriptase stalling assay. Adducts, shown as an orange ball, stop the movement of reverse transcriptase resulting in RNA that is not reverse transcribed. This can be detected by qPCR of the resulting cDNA template; lower signal signifies more adducts. Figure in part generated in BioRender: https://BioRender.com/d61e008. (**E**) Top, timeline of the drug treatments of HCT116 cells for this analysis, which are the same as in panel (B). Bottom, graph depicting the levels of a qPCR amplicon in the 18S ribosomal RNA (rRNA) after oxaliplatin treatment compared to vehicle treatment set to 1. Significance was calculated by ANOVA using Šídák multiple comparison testing. (**F**) Top, schematic of platinum agent treatment of HCT116 cells and analysis, which is as in panel (C). Bottom, analysis of adduct formation after oxaliplatin, cisplatin or carboplatin treatment. Statistical significance, or lack thereof, was determined by ANOVA using Šídák multiple comparison testing. *P < .05, **P < .01.

Having shown that the antimetabolite 5FU causes direct RNA damage, we asked whether further cytotoxic chemotherapies do likewise. First, we analysed the pseudo-alkylating drug oxaliplatin and its sister compounds cisplatin and carboplatin. We adapted our AquIRE method using a different antibody that has previously been used to detect platinum adducts on DNA (Fig. 2A). The antibody has been shown to bind to all the platinum agents as adducts on DNA [32, 33], though whether binding affinity is equivalent for all agents is unknown. First, we detected oxaliplatin covalently bound to RNAs after a 6-h dose of 50 µM (Fig. 2B). A lower, clinically achievable dose of 2.5 µM did not induce detectable adducts at this time but did result in adducts after 24 h of treatment (Fig. 2C). In contrast, neither cisplatin nor carboplatin were able to induce consistent, detectable adducts on RNA after prolonged exposure (Fig. 2C). Thus, oxaliplatin, but not cisplatin or carboplatin, cause direct damage to RNA that can be detected by AquIRE. At clinically achievable doses of oxaliplatin this damage occurs only after 6 h, in contrast to the rapid damage detected following 5FU treatment (Fig. 1E).

To confirm drug-induced RNA damage with an alternative, transcript-specific method, we adapted a previously published cDNA stalling methodology [34] (Fig. 1D). Here, adducts on RNA block reverse transcriptase, resulting in loss of cDNA that is detected by qPCR. Following reverse transcription with random hexamers, we designed qPCR primers to amplify known solvent-exposed regions of the abundant 18S rRNA. Consistent with the AquIRE results for total RNA damage, the levels of 18S cDNA were dramatically reduced by oxaliplatin treatment (Fig. 2E). Importantly, this reduction in qPCR signal is not a result of reduced RNA integrity (Supplementary Fig. S2A). Thus, the loss of 18S cDNA can be attributed to oxaliplatin adducts and not to other effects on 18S rRNA integrity. We do observe that 24 h of oxaliplatin treatment reduces total RNA content per cell, likely due to disruption to nucleoli and rRNA synthesis [35, 36] (Supplementary Fig. S2B). Nevertheless, the effect measured in our qPCRs are not solely due to reduced rRNA content per cell as these assays are controlled by using the same rRNA input by mass. Corroborating the specificity of RNA damage caused by oxaliplatin, the reverse transcription stalling method showed that oxaliplatin, but not cisplatin or carboplatin, reduced the 18S rRNA signal (Fig. 2F). The two methods, AquIRE and cDNA stalling, also aligned perfectly for the time taken for measurable RNA damage to occur following oxaliplatin treatment. Notably, there is high variability in the detected 18S rRNA adducts after 24-h incubation with cisplatin or carboplatin (Fig. 2F). We attribute this to the multi-targeting nature of the drugs, meaning that in some instances 18S rRNA damage occurs to a higher extent than others.

### Reversible changes in RNA methylation induced by temozolomide

**Figure 3.**
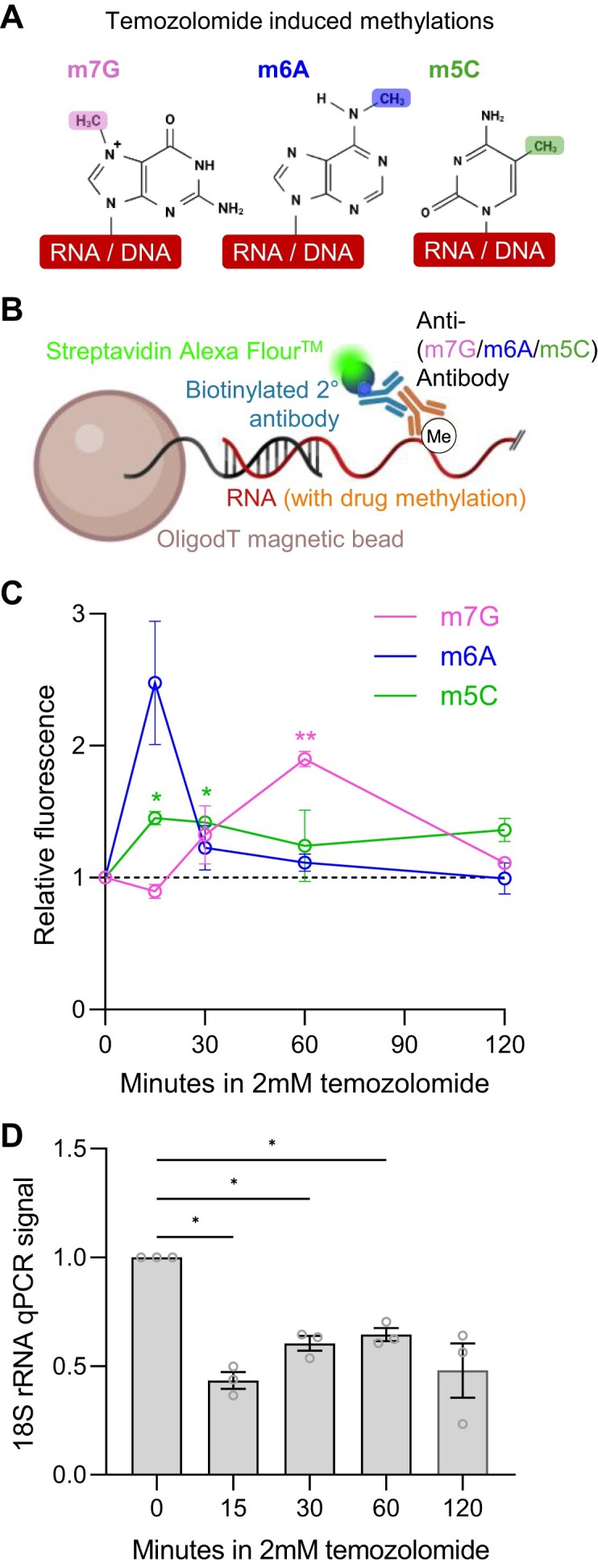
Temozolomide causes reversible changes in the epitranscriptome. (**A**) Representation of the effects of temozolomide on DNA and its hypothesized effect on RNA—the methylation of m7G, m6A, or m5C. Figure was partly generated in BioRender: https://BioRender.com/g55d965. (**B**) Schematic of the AquIRE detection method for temozolomide-induced RNA damage. (**C**) A172 cells were treated with 2 mM temozolomide for the indicated durations, RNA extracted and the relative abundance of m7G, m6A, and m5C plotted compared to vehicle treatment set to 1. Data show the average of three biological replicates ± SEM for each methylation. Significance compared to vehicle was calculated by ANOVA with Šídák multiple comparison testing. (**D**) RNA extracted from A172 cells after the indicated times in 2 mM temozolomide was reverse transcribed and analysed by the cDNA stalling analysis. qPCR of the 18S rRNA amplicon are plotted for three biological replicates ± SEM. Significance compared to vehicle was calculated by ANOVA with Šídák multiple comparison testing. *P < .05, **P < .01.

Temozolomide is used for the treatment of glioblastoma due to its ability to cross the blood-brain barrier. Within the body, temozolomide activation results in a highly reactive methyldiazonium ion that directly methylates biomolecules [37]. In DNA this occurs primarily at the N7-position of guanosines (m7G) with very little evidence for other methylation sites, such as the C5-position methylation of cytosine (m5C) or the N6-position of adenosine (m6A) [38]. However, whether and in what proportion these positions are methylated in RNA is unknown. Thus, using antibodies specific to m7G, m6A, and m5C, we adapted our AquIRE platform to detect temozolomide-induced RNA damage (Fig. 3A and B). Using RNA extracted from A172 glioblastoma cells with and without temozolomide treatment, we saw induction of each of these methylation events with temporal specificity (Fig. 3C). m6A occurs rapidly then drops sharply, m5C occurs rapidly but is retained, and m7G is the latest to occur and is transient (Fig. 3C). Methylation of RNA by temozolomide was confirmed by LC-MS analysis, where induction of m6A, m5C, and m7G was each observed after 30 min of treatment (Supplementary Fig. S3A). Unlike AquIRE, LC-MS quantification was extremely variable between biological replicates. Each of the three replicates analysed by LC-MS showed at least a twofold induction of one or more of these methylations. Induction of m5U, for which no antibody is available, was also observed in two of the replicates (Supplementary Fig. S3A). Methylation of RNA also impairs reverse transcription [39], allowing us to read out temozolomide-induced RNA damage using the cDNA stalling assay. qPCR for the 18S rRNA shows rapid and sustained RNA damage, illustrated by a reduction in signal (Fig. 3D). This is consistent with methylation induction seen by LC-MS of modifications known to stall reverse transcription (Supplementary Fig. S3B). Specifically, temozolomide induced methylation of the 2′O position of the sugar backbone or positions on the Watson–Crick face. Thus, temozolomide treatment results in methylation of RNA, which changes dynamically over time. This implies that each methylation site has (i) specific susceptibility to drug-induced methylation and (ii) specific mechanisms to reverse the methylation.

### AquIRE detects endogenously written RNA modifications

We have shown that AquIRE can detect covalent RNA damage caused by three different chemotherapies. The chemical modifications of RNA directed by writer enzymes expand the coding and functional capability of RNA, being termed the epitranscriptome [40]. We expected AquIRE would be able to capture these epitranscriptomic markers and therefore tested our method using m6A and pseudouridine

**Figure 4.**
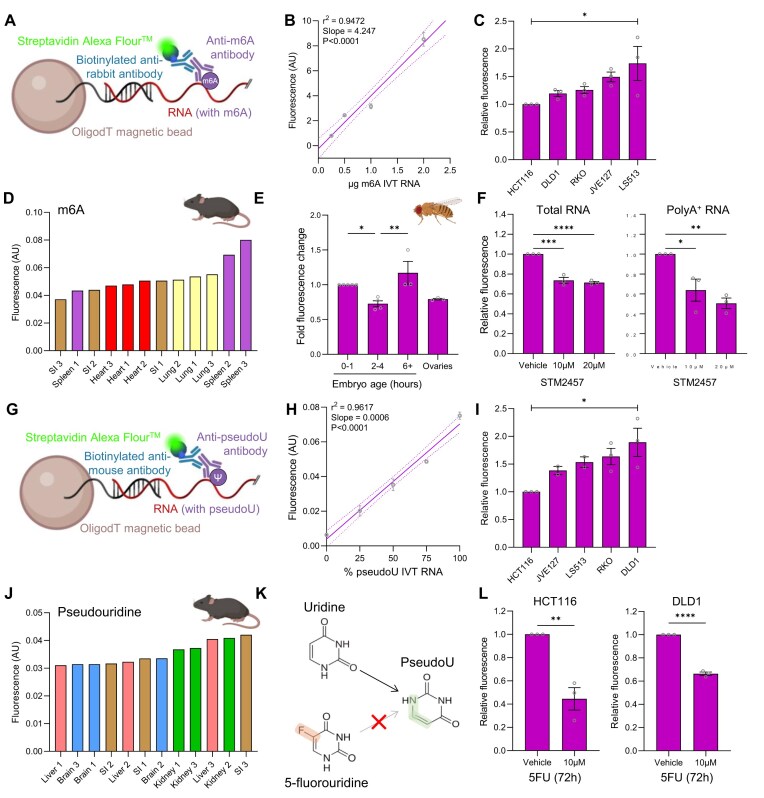
AquIRE sensitively quantifies the epitranscriptome across species. (**A**) Schematic of the detection of m6A using the AquIRE protocol. (**B**) Different amounts of IVT RNA with 50% m6ATP were analysed by AquIRE. Mean fluorescence reads ± SEM from three technical replicates were plotted against the quantity of m6A. Purple line plots a simple linear regression, dashed lines are the 95% confidence interval. Details of the linear regression fit are inset. (**C**) RNA was isolated from five CRC cell lines and analysed for m6A content. Values are expressed as the mean of three biological replicates ± SEM relative to the HCT116 cell line. Significance was tested by ANOVA. (**D**) AquIRE determined m6A levels in RNA samples from four tissues from three mice. Colour coded bars represent a tissue with the numbers in the annotation indicating the same animal. Values are presented as the raw fluorescence read from one technical replicate per tissue per animal. (**E**) RNA was extracted from *Drosophila* embryos at the indicated timepoints or adult ovary tissue then m6A levels quantified by AquIRE. The graph plots the fold fluorescence change compared to the 0–1 h timepoints from at least three biological replicates. Significance compared to 2–4 h timepoint was tested using an ANOVA with Šídák multiple comparison testing. (**F**) RNA was extracted from HCT116 cells following treatment with STM2457 for 24 h at the indicated concentrations. The graphs plot relative fluorescence compared to vehicle for total RNA, left, or polyA RNA, right. Data are from three biological replicates and show the mean ± SEM. Significance compared to vehicle was tested using an ANOVA with Šídák multiple comparison testing. (**G**) Schematic of the detection of pseudouridine (Ψ) with the AquIRE protocol. (**H**) IVT RNAs made with the indicated percentage of pseudoUTP were analysed. Mean fluorescence reads ± SEM from three technical replicates were plotted against the quantity of Ψ. Purple line plots a simple linear regression, dashed lines are the 95% confidence interval. Details of the linear regression fit are inset. (**I**) RNA was isolated from five CRC cell lines and analysed for Ψ content. Values are expressed as the mean of three biological replicates ± SEM relative fluorescence compared to HCT116. Significance was tested by ANOVA. (**J**) AquIRE was used to determine Ψ levels in RNA samples from four tissues from three mice. Data are represented as in panel (D) above. (**K**) Schematic indicating that 5FUridine cannot be converted to pseudouridine. The modified part of the Ψ base is shown in green and the hindering fluorine atom in 5FUridine in red. (**L**) RNA was extracted from HCT116 or DLD1 cells following treatment with 5FU at 10 µM for 72 h. The graphs show AquIRE data plotted as relative fluorescence compared to vehicle treatment. Data are from three biological replicates and show the mean ± SEM. For both cell lines, significance was tested using an unpaired t test. *P < .05, **P < .01, ***P < .001, ****P < .001.

Using a commercially available m6A antibody (Fig. 4A), we found that the AquIRE platform sensitively detected m6A levels (Fig. 4B). Utilizing this sensitivity, we asked whether consistent differences in m6A between biological samples could be detected, doing this for total RNA extracted from five different colorectal cancer cell lines (Fig. 4C) and RNA from four tissues from three mice (Fig. 4D). We were able to detect consistent differences in m6A levels between the cell lines and observe global differences in this epitranscriptomic marker across different mouse tissues. Next, we used a *Drosophila* model of embryo development, where an increase in m6A levels had previously been observed using mass spectrometry during the transition from maternal to zygotic transcription [41]. We recapitulated this data with AquIRE, observing an increase in m6A levels at 6 h compared to pre-transition timepoints (Fig. 4E). This demonstrates the sensitivity of AquIRE to rapidly detect the epitranscriptome from *in vivo* samples. Finally, we used colorectal cancer cells treated with an inhibitor of the m6A-writer METTL3, STM2457, to analyse m6A levels in total RNA and messenger RNA . AquIRE detected a reduction in m6A in total RNA following METTL3 inhibition (Fig. 4F). Omitting the *in vitro* polyA tailing step from our protocol allowed analysis of endogenous polyA RNA, where an even greater reduction was seen (Fig. 4F).

To assess pseudouridylation we used a commercially available antibody in our AquIRE protocol (Fig. 4G), finding a highly sensitive correlation of signal to pseudouridine content (Fig. 4H). Using the same panel of colorectal cancer cell lines and four tissues from three mice we were able to measure relative pseudouridine levels with sufficient accuracy to infer significant differences for the cell lines. (Fig. 4I and J). Finally, 5FU incorporation into RNA has previously been shown to reduce pseudouridine levels, due to the inability of pseudouridylating enzymes to chemically modify a fluorinated substrate (Fig. 4K) [42]. An AquIRE analysis of RNA extracted from two colorectal cancer cell lines treated with 5FU supports these previous findings and demonstrates the ability of our method to detect epitranscriptomic modulation (Fig. 4L).

### AquIRE sensitively detects cellular and cell-free glycoRNAs

**Figure 5.**
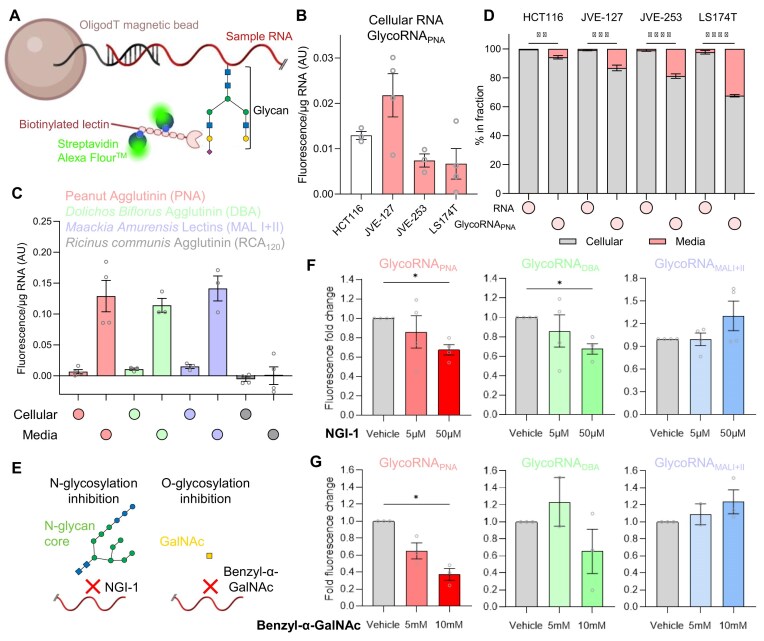
AquIRE detects glycoRNAs formed by N- and O-glycosylation. (**A**) Schematic of the AquIRE methodology to detect glycoRNAs using biotinylated lectins. (**B**) RNA extracted from four colorectal cancer cell lines was analysed for glycoRNA levels using the PNA lectin—denoted as the glycoRNA_PNA_ signal. Values are plotted as the fluorescence per µg of RNA from three biological replicates ± SEM after normalization against IVT RNA. HCT116 cells (white bar) are non-mucinous in origin, while the other cell lines (red bars) are mucinous. (**C**) RNA from LS174T cells and growth media was isolated and analysed for glycoRNA expression using four different lectins. Values are presented as the fluorescence per µg of RNA normalized against IVT RNA from at least three biological replicates. (**D**) RNA was extracted and quantified from four different cell lines and their growth media, then the distribution of RNA plotted for three biological replicates (left bar per cell line). The glycoRNA_PNA_ content of the same RNA samples was quantified, and the distribution of signal normalized against RNA content and plotted as a percentage. Significance was determined independently for each cell line by two-way ANOVA with Šídák multiple comparison testing. (**E**) Schematic representation of the experimental approaches to inhibit N- and O-glycosylation using NGI-1 and Benzyl-α-GalNAc, respectively. (**F**) LS174T cells were treated with the N-glycosylation inhibitor NGI-1 for 24 h at the indicated concentrations, and RNA isolated from cells. GlycoRNA_PNA_, glycoRNA_DBA_, and glycoRNA_MALI+II_ levels were detected by AquIRE and expressed as the fold change in fluorescence normalized against IVT RNA compared to vehicle treatment. Significance compared to vehicle was tested using an ANOVA with Šídák multiple comparison testing. (**G**) LS174T cells were treated with the O-glycosylation inhibitor Benzyl-α-GalNAc for 48 h at the indicated concentrations, RNA isolated and the same AquIRE analyses performed as in panel (F). Data are presented and significance determined as in panel (F). *P < .05, **P < .01, ***P < .001, ****P < .001.

GlycoRNAs are a class of heterogeneous RNAs that are covalently modified with glycan moieties and expressed on mammalian cell surfaces [22]. They have known functions in neutrophil recruitment and cell attachment [24, 25], while upon cell surfaces they enable peptide entry via interaction with specific RBPs [43]. However, simple questions regarding glycoRNA conservation among species and non-cell surface localization remain to be answered. We reasoned that the AquIRE platform could address these without the need of orthogonal labelling reagents [22] or covalent modification of glycoRNA moieties [44] through the use of glycan-binding lectins in place of the antibody approach used thus far. Therefore, we modified the AquIRE protocol using biotin-tagged lectins (Fig. 5A) and were able to detect a consistent and significant fluorescent signal using RNA isolated from four different colorectal cancer cell lines (Fig. 5B). We used the lectin peanut agglutinin (PNA) for this initial detection and gained similar results with two alternative lectins, *Dolichos Biflorus* agglutinin (DBA) and the *Maackia Amurensis* lectins (MAL I + II) (Fig. 5C). This was lectin-specific, as *Ricinus communis* agglutinin (RCA_120_) gave no increase in fluorescence compared to glycan-free IVT RNA. For glycoRNA detection by AquIRE, we set a nomenclature based on the specific lectin, for example with PNA we detect glycoRNA_PNA_. Binding of PNA or DBA is stabilized by divalent cations which are absent from our analysis. To confirm that both lectins can bind to their glycan targets in our assay conditions, we performed competitive elution assays with soluble galactose-1-phosphate for PNA and N-acetyl-D-galactosamine for DBA (Supplementary Fig. S4A). In both cases applying these soluble sugars efficiently eluted the fluorescent signal. This demonstrates that our assay conditions are amenable for binding of PNA and DBA lectins to glycans, supporting the existence of glycoRNA_PNA_ and glycoRNA_DBA_.

Given similarities in their glycan biogenesis [22], we asked whether glycoRNAs share the spatial distribution of glycoproteins. Cell-free glycoproteins are well characterized, while cell-free glycoRNAs have only recently been reported [45]. To address this, we analysed the RNA and glycoRNA content of the growth media from cultures of four colorectal cancer cell lines. Three of these lines, JVE-127, JVE-253, and LS174T, originated from the mucinous subtype of colorectal cancer characterized by copious levels of cancer-cell derived mucus. This mucus is made of O-glycosylated mucin proteins, with elevated production of these glycoproteins retained in culture. In comparison, the HCT116 cell line is not mucinous and produces orders of magnitude less glycosylated mucins. Quantifying the distribution of glycoRNA_PNA_ signal adjusted for RNA content in each fraction and total RNA (calculated as the yield from extraction), we consistently found a greater fraction of glycoRNAs in the cell-free fraction than the distribution of total RNA (Fig. 5D). Similar cellular and extracellular glycoRNA levels would suggest their distribution is not actively controlled. The enrichment we observe, higher glycoRNA_PNA_ per µg of cell-free RNA, indicates an as yet unknown active mechanism of glycoRNA_PNA_ delivery into the extracellular environment. In line with this, the fraction of extracellular glycoRNA_PNA_ in the mucinous cell lines is consistently higher than the fraction in the non-mucinous cell line. Indeed, in LS174T cells 32% of glycoRNA_PNA_ is extracellular, compared to only 2.5% of total RNA. Furthermore, the glycoRNA_PNA_ fluorescence per µg of RNA is almost 20-fold higher in cell-free RNA than cellular RNA, an observation consistent for glycoRNA_DBA_ and glycoRNA_MALI+II_ in LS174T cells (Fig. 5C) and for glycoRNA_PNA_ across the panel of CRC cell lines (Fig. 5B and Supplementary Fig. S4B).

### RNA is subject to both N- and O-glycosylation

Having established that AquIRE can detect unlabelled glycoRNAs, we next sought to use this technology to confirm and extend previous observations regarding glycoRNA synthesis. To do so we used RNA from the LS174T colorectal cancer cell line. First, we incubated purified cellular and extracellular RNA with the N-glycosylase PNGase F, which was previously shown to remove glycoconjugates from RNA [22]. In agreement, we observed a reduction in glycoRNA_PNA_ signal from both cellular and cell-free RNA following incubation (Supplementary Fig. S4C). However, the reduction in signal was not complete, implying that a fraction of glycoRNA_PNA_ is not derived from N-glycosylation. To understand this in more detail, we leveraged the distinct binding specificities of the lectins used here (Supplementary Fig. S4D). Lectins bind tightly to sugar structures, demonstrating preferences for certain structures over others [46]. MAL II was previously shown to bind to glycoRNAs [22, 25] and preferentially binds to sialylated glycans. These can be found on both N- and O-glycosylated substrates. N-glycosylated protein substrates are less likely to have sugar structures bound by either PNA or DBA. Both of these lectins preferentially bind to terminal glycans commonly found on O-glycosylated proteins across multiple species [46]. This leads to the hypotheses that either N-glycans on RNA can contain sugars with affinity for PNA or DBA and/or RNAs are substrates for O-glycosylation. Consistent with both hypotheses, two preprints have independently demonstrated that N-glycans in glycoRNAs contain the T antigen bound by PNA [47] and that O-glycosylation indeed occurs on RNA [48]. Furthermore, glycoRNAs labelled with Ac_4_GalNAz, almost exclusively synthesized into O-glycoproteins, were found to be more abundant than those labelled with Ac_4_ManAz in multiple cell lines [49]. Ac_4_GalNAz was also recently observed in cellular and extracellular vesicles [45]. Notably, in the same study, Ac_4_GalNAz signal in RNA was dramatically reduced upon N-glycosylation inhibition.

To investigate this further with AquIRE, we treated cells with inhibitors of either N- or O-glycosylation (Fig. 5E). To block N-glycosylation, we used the oligosaccharyltransferase inhibitor NGI-1, which is known to inhibit the N-glycosylation of RNAs [22]. For O-glycosylation we used the inhibitor Benzyl-α-GalNAc, which acts as a decoy substrate for the initiating GalNAc in protein O-glycosylation [50] and potentially RNAs. Following each drug treatment, we used AquIRE to detect glycoRNA_PNA_, glycoRNA_DBA_, and glycoRNA_MALI+II_. Notably, glycoRNA_PNA_ is decreased by inhibition of either N- or O-glycosylation (Fig. 5F and G). GlycoRNA_DBA_ is dependent on N-glycosylation, with a reduction seen upon NGI-1 treatment, while there was no reduction upon inhibition of O-glycosylation (Fig. 5F and G). We find that glycoRNA_MALI+II_ levels are insensitive to either drug treatment (Fig. 5F and G).

Overall, using the sensitivity of our AquIRE assay in conjugation with lectins allows us to detect glycoRNAs that display similar molecular biology to those described previously. We also identify cell-free glycoRNAs in colorectal cancer models. Furthermore, we demonstrate that different lectins are reactive to glycans that can be reduced by inhibition of N- or O-glycosylation. This confirms the O-glycosylation of RNAs while highlighting discrepancies between glyco-conjugation of protein and RNA substrates. Consistent with this, GlycanDIA—a data-independent acquisition glycomics platform—benchmarked released N-glycans on RNA and proteins from the same biological sources [51]. In the models analysed, >25% of the glycan structures found on RNA are not present on protein, leading to the conclusion that the biosynthetic pathways for glycoRNAs and glycoproteins indeed differ. It is therefore important not to conflate knowledge of protein glycosylation with the unknowns of glycoRNA synthesis before the latter is formally analysed.

### GlycoRNAs are expressed across the kingdoms of life

**Figure 6.**
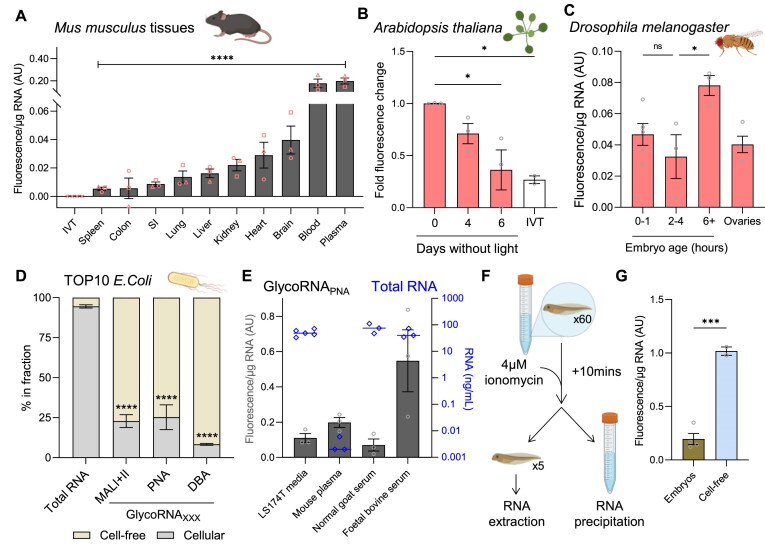
GlycoRNAs can be cell-free and are found in all domains of life. (**A**) RNA was extracted from the indicated tissues from three different mice (each a different symbol) and analysed by AquIRE for glycoRNA_PNA_. Graph shows the mean fluorescence per µg of RNA for each tissue, which was normalized against IVT RNA set to 0 (also shown). Significance was determined by ANOVA analysis. (**B**) RNA was extracted from *Arabidopsis thaliana* Col-0 ecotype leaves that had been grown in the absence of light for the indicated times. AquIRE detected glycoRNA_PNA_ content in biological triplicates of these RNA samples compared to an equivalent mass of IVT RNA. The fold change in raw fluorescence reads is plotted compared to the 0-day timepoint. Significance was determined by ANOVA with Šídák multiple comparison testing. (**C**) RNA was extracted from *Drosophila* (*y*^1^*w*^67c23^) embryos at the indicated timepoints or adult ovary tissue and glycoRNA_PNA_ content determined by AquIRE. Graph plots the mean fluorescence per µg for each sample from at least three biological replicates. Within the embryo samples, significance compared to 2–4 h timepoint was tested using an ANOVA with Šídák multiple comparison testing. (**D**) RNA from *E. coli* (TOP10) cells and growth media was isolated and quantified, then the distribution of RNA plotted for four biological replicates (left). The glycoRNA_PNA_, glycoRNA_DBA_, and glycoRNA_MALI+II_ levels were quantified, then the distribution of signal normalized against RNA content and plotted as a percentage. Significance was determined for each glycoRNA type by two-way ANOVA with Šídák multiple comparison testing. (**E**) RNA was extracted from the media of LS174T cells, mouse plasma and serum from goat and cow. The grey bars plot the mean glycoRNA_PNA_ fluorescent signal per µg of RNA, while the blue bars plot the RNA content of each liquid sample in ng/ml. (**F**) Ionomycin secretagogue protocol for isolation of whole organism and cell-free *Xenopus laevis* RNA. Embryos were treated for 10 min with ionomycin, then RNA extracted from pooled whole embryos or their growth media. (**G**) RNA samples as in panel (C) were analysed for glycoRNA_PNA_ content by AquIRE. Graph plots the mean fluorescence per µg of RNA from four pooled embryo samples and two cell-free samples. Multiple figure panels were generated in BioRender: https://BioRender.com/t94t009. Significance was determined by unpaired t test. *P < .05, ***P < .001, ****P < .001.

Next, we leveraged the ability to measure glycoRNAs in any cell, tissue, or liquid sample to identify which species synthesize glycoRNAs. First, we sampled nine tissues and blood plasma from wild-type mice and quantified the relative glycoRNA_PNA_ expression normalized to IVT RNA and expressed per µg of input RNA. GlycoRNA_PNA_ could be detected in all tissues and within blood plasma, with more than an order of magnitude between the highest expressing tissue, blood, and the lowest, spleen (Fig. 6A). This tissue specificity likely indicates distinct functions that are yet to be revealed. The RNA recovery at the end of these AquIRE experiments varied by tissue, from over 100% down to 40% (Supplementary Fig. S5A). The reasons for this are unclear but may relate to the efficiency of polyadenylation prior to immobilization of RNA on oligodT beads. Importantly, there was not a positive correlation between fluorescent signal and RNA recovery (Supplementary Fig. S5B).

To date, glycoRNAs have only been described in mammals. We therefore sought to assess the conservation of these molecules by analysing their expression in diverse organisms and models of specific biology, from embryo development to senescence. Throughout, we maintain a focus on the expression of glycoRNAs in cellular and cell-free samples. First, we found that the model plant organism, *Arabidopsis*, expresses glycoRNA_PNA_, both as a seedling and in leaves (Supplementary Fig. S5C and D). Using an *Arabidopsis* model of leaf senescence, we found that glycoRNA_PNA_ levels were significantly reduced during senescence and essentially absent in leaves deprived of light for 6 days (Fig. 6B). This is a clear demonstration of dynamic changes in glycoRNAs levels in response to a physiological stimulus. Next, we used *Drosophila* embryos to determine the levels of glycoRNAs during embryonic development and in the ovary. The transition from maternal to zygotic transcription occurs in two waves between 1 and 3 h of development [52] and has been correlated with changes in RNA modifications such as m6A [41]. GlycoRNA_PNA_ was present at all timepoints in our analysis, as well as in adult fly ovaries (Fig. 6C). The presence of glycoRNA_PNA_ in the 0–1 h embryos, prior to the onset of zygotic transcription, shows that they are maternally deposited. In addition, there is a significant increase in glycoRNA_PNA_ in older stage embryos, showing that glycoRNAs are abundant during embryogenesis (Fig. 6C). These fundamental observations in plant and insect models expand the horizons of where and when glycoRNAs are expressed. Furthermore, using the sensitivity of AquIRE allows us to outline physiological settings where glycoRNA_PNA_ expression is modulated.

To evaluate the species conservation of glycoRNAs further we asked if the single-celled eukaryote model, *S. cerevisiae* expresses glycoRNA_PNA_. We found expression in both *S. cerevisiae* cells and in cell-free media extracts (Supplementary Fig. S5E). Significantly more glycoRNA_PNA_ was detected in the growth media than total RNA, leading to the conclusion that *S. cerevisiae* actively partitions glycoRNA_PNA_ preferentially towards their environment. In a similar analysis, we asked if the prokaryotic model organism, *E. coli*, expresses glycoRNAs within cells and in its environment. We found glycoRNA_PNA_, glycoRNA_DBA_, and glycoRNA_MALI+II_ present in both sample types, with an enrichment of cell-free RNA compared to cellular RNA in each case (Fig. 6D). Indeed, we observe >70% of glycoRNA detected with each lectin are cell-free in this *E. coli* model. GlycoRNA_PNA_, glycoRNA_DBA_, and glycoRNA_MALI+II_ were also detected in commercially sourced cellular RNA extracted from the K-12 strain of *E. coli* (Supplementary Fig. S5F). Notably, the expression of glycoRNA_PNA_ and glycoRNA_DBA_ between the two bacterial strains was comparable, but the K-12 strain had higher levels of glycoRNA_MALI+II_ per µg of RNA (Supplementary Fig. S5F). The reason for this is unclear but may indicate heterogeneity between engineered strains in their glycoRNA synthesis and expression.

Having observed glycoRNAs present in the blood plasma of mice (Fig. 6A), we analysed the expression of RNA and glycoRNA_PNA_ in commercially available mammalian serum products from *Capra aegagrus hircus* (normal goat serum) and *Bos taurus* (foetal bovine serum). RNA was present in both cell-free liquids, consistent with previous reports [53] and with similar or higher glycoRNA_PNA_ expression per µg of RNA to that seen in media conditioned by LS174T cells (Fig. 6E). To further investigate the cell-free expression of glycoRNAs, we used *Xenopus tropicalis* embryos in an induced secretion model using the secretagogue ionomycin. RNA was extracted from whole embryos or their cell-free environment after ionomycin treatment and used to detect glycoRNA_PNA_ (Fig. 6F). We were able to detect high expression of glycoRNA_PNA_ in both samples, with the cell-free samples showing a significantly higher signal per µg of RNA than embryos (Fig. 6G). Of note, *Xenopus* embryos showed the highest expression of glycoRNA_PNA_ per µg of RNA across the >20 samples from organisms or cells analysed here (Supplementary Fig. S6A). Meanwhile, the media in which *Xenopus* were treated with ionomycin showed the highest glycoRNA_PNA_ per µg RNA of any of the >10 cell-free sample analysed here (Supplementary Fig. S6A).

### GlycoRNAs are present in human tumours and determine chemotherapy responses

**Figure 7.**
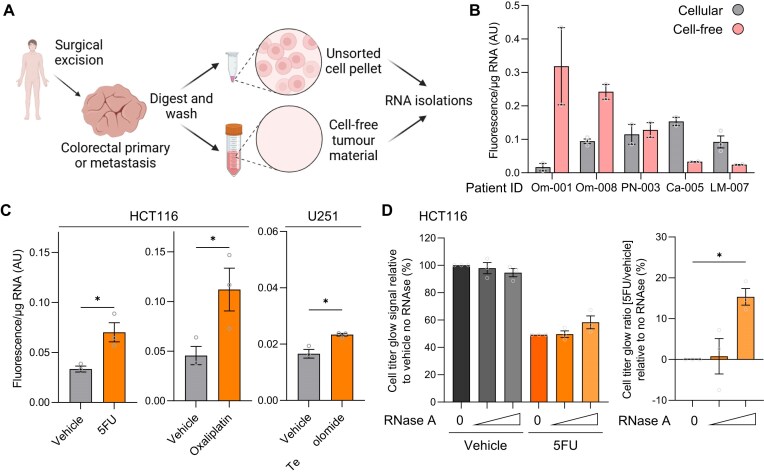
GlycoRNAs are elevated by and determine response to RNA-damaging chemotherapy. (**A**) Schematic of colorectal tumour processing to generate unsorted cell pellets and cell-free tumour material. RNA was extracted from each fraction for analysis. Figure generated in BioRender: https://BioRender.com/s88×264. (**B**) RNA from panel (A) was analysed for glycoRNA_PNA_ by AquIRE and plotted as the fluorescence per µg of RNA for five biologically independent tumours, showing the mean of at least two technical replicates. The *x-*axis lists the unique tumour name. (**C**) HCT116 colorectal cancer cells were treated with 10 µM 5FU for 72 h, 50 µM oxaliplatin for 6 h, or their vehicles, and U251 glioblastoma cells were treated with 2 mM temozolomide or vehicle for 2 h. GlycoRNA_PNA_ content was determined in RNA extracted after these treatments, with the graphs plotting the mean fluorescence per µg from three biological replicates per drug ± SEM. Significance was determined by unpaired *t-*test. (**D**) HCT116 cells were treated with 10 µM 5FU for 72 h in the presence of 100 or 200 µg/ml RNase A or no enzyme. Right, the change in Cell Titer Glo viability analysis signal was plotted relative to vehicle with no enzyme set to 100%. Due to variability in the efficacy of 5FU between biological replicates (5% SEM) values after drug treatment were normalized against the average effect of 5FU in the absence of enzyme (49.3%). Right, the ratio of the cell viability signal of 5FU/vehicle for the three different enzyme conditions was plotted as a percentage change in viability compared to no-enzyme set to 0. Data represent the mean of three biological replicates ± SEM. Significance compared to no-enzyme condition was tested using an ANOVA with Šídák multiple-comparison testing. *P < .05.

Finally, we were interested in the role of glycoRNAs in disease. We therefore applied our AquIRE methodology to detect glycoRNAs from clinical samples, focusing on glycoRNA expression in the tumours of colorectal cancer patients. Following surgical excision, five tumours were processed to yield a paired unsorted cell pellet and cell-free tumour material (Fig. 7A). RNA was extracted from both sources and analysed for glycoRNA_PNA_ content expressed as fluorescence/µg RNA. GlycoRNA_PNA_ was detected at varying levels across all 10 samples (Fig. 7B). GlycoRNA_PNA_ expression displayed intra- and inter-tumour heterogeneity across patient samples. When comparing the same sample type between different patients, glycoRNA_PNA_ levels differed by an order of magnitude. However, between sample types for individual patients, some displayed similar expression, while others had large differences. These data show that glycoRNAs, such as glycoRNA_PNA_, are likely ubiquitously expressed in, and released by tumours in patients. Our next analyses asked whether these glycoRNAs are regulated or functional.

Linking back to our analysis of drug-induced RNA damage, we asked whether specific chemotherapies could modulate the levels of glycoRNAs in disease-specific cell line models. Thus, we treated the HCT116 cell line with 5FU or oxaliplatin and observed a consistent increase in glycoRNA_PNA_ expression (Fig. 7C). Similarly, treating the glioblastoma cell line U251 with temozolomide also resulted in an increase in glycoRNA_PNA_ expression (Fig. 7C). We have shown that RNA from HCT116 cells is damaged by either 5FU or oxaliplatin (Figs 1 and 2), while U251 RNA demonstrates m7G accumulation after temozolomide treatment (Supplementary Fig. S6B). This provides further evidence for glycoRNAs being dynamically modulated, adding to the data from the plant and fly models presented above.

Finally, we asked whether there is a role for glycoRNAs in relation to chemotherapy response in colorectal cancer cells. To functionally test this, we aimed to remove cell surface and extracellular RNAs using RNase A, similar to the experiments performed previously [22, 24, 25]. Notably, we found that RNase A did not significantly reduce the RNA content in cell culture media but did reduce the glycoRNA_PNA_ levels (Supplementary Fig. S6C). The RNase A resistance of the cell-free RNA in this experiment may be attributable to protection by RNA-binding proteins or within membranous bodies. The use of RNase A allows us to ask what effect ∼35% reduction in glycoRNA_PNA_, and potentially other glycoRNA species, has on response to chemotherapy.

We reasoned that glycoRNAs may facilitate uptake of 5FU due to the ability of the nucleoside to base-pair with adenosine bases in the RNA. Consistent with this, a recent study demonstrated cell-surface RNAs are required for cell entry of the TAT viral peptide [43]. We incubated HCT116 cells in culture with and without RNase A added to their media. RNAse A likely also digests cell surface RNA, which we did not measure. We performed these digests with and without treatment with 5FU and asked how this affected cell viability. RNase A had minimal effect on HCT116 cell viability (Fig. 7D). However, in the presence of 5FU, the RNase A-treated cells were significantly more viable than undigested controls (Fig. 7D). Thus, cell-free (or cell surface) glycoRNA_PNA_ may be required for the maximum cytotoxicity of 5FU in HCT116 tumour cells. This reveals a previously unknown disease modulatory mechanism, attributable to glycoRNA, in determining cell responses to an RNA-damaging agent.

## Discussion

The cellular RNA damage responses are well documented and are beginning to be linked to normal processes and pathophysiology [3, 10, 11, 14, 17, 19]. Based on previous observations, we hypothesized that common chemotherapies cause RNA damage and set out to directly measure this biology. To do so, we developed the AquIRE research platform, measuring drug-induced RNA damage for 5FU, oxaliplatin, and temozolomide. This allowed us to document their previously unknown temporal dynamics and multifaceted mechanisms of action. The temporal differences between the compounds are notable, with 5FU and temozolomide able to induce RNA damage rapidly (<30 min), while clinically achievable doses of oxaliplatin require >6 h to induce detectable damage. This is likely linked to each compound’s mechanisms of biochemical activation and metabolism. Given that oxaliplatin and 5FU are often administered in sequential combination, it is notable that oxaliplatin is dosed prior to 5FU. The temporal dynamics observed here suggest that this may allow the RNA damage induced by both compounds to occur concomitantly, which may enhance cytotoxicity.

For temozolomide and 5FU, we observe transient inductions of RNA damage, implying cell-intrinsic mechanisms to reverse damage or degrade damaged transcripts. An ASCC-ALKBH3 repair pathway has been previously described for the repair of alkylation, such as that caused by temozolomide [16], and activation of ribosome quality control has been observed upon 5FU treatment [28]. Understanding how damaged RNA transcripts are dealt with will potentially reveal the cause of the transient RNA damage observed here. Our work presents three examples of antimetabolite and alkylating compounds, prompting questions regarding the extent and dynamics of RNA damage by clinically used compounds from these same classes.

The long-accepted mechanism of action for 5FU is the inhibition of thymidylate synthase via the formation of a covalent complex with a 5FU metabolite [54]. Challenging this assumption, the importance of 5FU incorporation into RNA and how RNA damage determines the cellular response to 5FU was recently published [5, 8]. This is consistent with previous reports linking 5FU incorporation into RNA to cytotoxicity from the 1980s [6, 7]. In truth, 5FU administration has both effects at the same time (affecting DNA synthesis and RNA metabolism and function), and both will contribute to cytotoxicity. We observe that 5FU impacts the epitranscriptome, via pseudouridine, at clinically achievable doses in colorectal cancer cell lines. This adds an additional layer to our understanding of how 5FU functions, with incorporation into RNA presenting opportunities to impact the epitranscriptome. Exemplifying this, a recent study demonstrates a sustained inhibition of both pseudouridine and methyluridine even after removal of 5FU [55]. Our work indicates that direct targeting of RNA or its epitranscriptome, two areas of active drug development during the 21st century [56–58], have likely been contributing to clinical benefit from 5FU for decades. Learning from classic chemotherapies may identify where to apply technological advances for specific RNA targeting in the future and thereby reduce collateral effects.

Lectins bind their dominant glycan targets with high affinity. However, it should be noted that they can bind alternative glycans with lower affinity, as demonstrated by a glycan microarray coupled to deep learning [46]. Lectin microarrays have demonstrated that the cell surface of *E. coli* contains binding sites for the lectins found to bind their RNAs in this work – PNA, DBA and MAL [59]. Binding of PNA to the cell surface of *S. cerevisiae* was not seen in parallel studies [60]. Thus, how PNA-binding sugars are found in this model is unclear. Direct RNA galactosylation was recently characterized in eukaryotic tRNAs [61]. Although this previous work found that *S. cerevisiae* lacks the enzymes required for this modification, it demonstrates the potential for discoveries at the convergence of glycobiology and RNA. We believe that measuring what sugars are present in a sample with a lectin-based approach has distinct advantages over bio-orthogonal sugar-based methods that depend on knowledge of glycan synthesis pathways, many of which remain to be defined in all species.

The use of IVT RNA as a negative control in our analyses of glycoRNAs shows that the lectins are indeed binding to conjugated glycans. The detection of glycoRNAs by AquIRE is further supported by the reduction in signal seen upon (i) incubation of RNA with glycan cleaving enzymes and (ii) following the use of small molecules that inhibit N- or O-glycosylation. Using AquIRE we identified glycoRNA expression in both the prokaryote and eukaryote domains of life and four of the seven kingdoms (bacteria, animals, plants, and fungi). As such, it appears likely that glycoRNAs are expressed in all life forms. In fact, throughout our studies we found only one condition where glycoRNAs were essentially absent – within the senescent leaves of *Arabidopsis thaliana*. Our work shows that inhibition of both N- and O-glycosylation reduced RNA glycosylation by some sugars but not by others. It remains to be seen if canonical O-glycoprotein glycans are present within N-glycans in RNA, or if inhibition of N-glycosylation impacts RNA stability and/or its O-glycosylation. Our work and others supports the notion that RNA glycosylation is different to canonical protein glycosylation [51], an area that merits further investigation. Expanding the repertoire of glycan detection using further lectins is an obvious approach to this. However, it should be noted that some lectins (not used here) have high background binding to IVT RNA, limiting their application in our assay.

The three canonical forms of glycosylation (O-, N-, and lipid) are conserved across the domains of life [62]. The conservation of glycoRNAs indicates that RNA does not hijack the enzymes of other biomolecules to become glycosylated but in all likelihood that these mechanisms of conjugation coevolved for RNA and protein substrates at the same time. It is important that we further understand the biochemistry of RNA glycosylation, given the impressive advances in structural studies to understand the exact mechanisms of protein glycosylation [63]. Perhaps the most obvious sites for O-glycosylation of RNA are the 2′OH of the ribose backbone or a 5′ or 3′ hydroxyl termini. The canonical bases within RNA lack hydroxyl groups, but these can be added by the action of enzymes or through oxidative damage, presenting potential attachment sites for O-glycans. O-glycosylation of a modified base would parallel the N-glycosylation site found on modified uridine bases [44].

Our observation of the conservation of glycoRNAs was only possible due to the AquIRE methodology with its key advantages of requiring low inputs and not relying on metabolic labelling. Unlike the use of bio-orthogonal agents, our method directly interrogates what the glycans are comprised of, independent of how they are made. For example, the original description of glycoRNAs using bio-orthogonal glycans found higher levels in mouse liver than spleen [22]. However, this assumes that the labelling reagent is available and metabolized in both organs in equal quantity. Our method does not rely on where an agent is metabolized within an organism, instead detecting the native glycoRNAs present in each location. Furthermore, the ability to switch lectins to analyse additional moieties will allow a rich picture of glycoRNA expression to be drawn. Our method is, however, limited to analysis of relative ensemble glycoRNA quantity between samples and does not provide detail of the number of glycans per RNA molecule. Future single molecule methods should allow us to understand if dynamic changes in glycoRNA levels are due to more or less transcripts being glycosylated or more or less glycosylation of the same number of transcripts.

Previous studies of glycoRNAs have documented their expression and function in various settings [22–25]. However, the modulation of glycoRNA levels in response to stimuli or during biological events has been less widely reported. Here we have shown that glycoRNA levels are dynamic in multiple settings, including during plant senescence, fruit fly development and in the response to RNA-damaging chemotherapies. This final observation was extended to find a functional role for glycoRNAs in determining response to the antimetabolite 5FU. Given that we observed a high degree of variability in glycoRNA expression in clinical samples, future studies should investigate how expression correlates with patient responses. The implication that tumour microenvironments that are rich in glycoRNA may promote chemotherapeutic cytotoxicity is also an exciting prospect with translational potential.

The application of the AquIRE method, as well as orthogonal methodologies, in this paper has allowed us to expand the horizons and functional dynamics of glycoRNAs across the kingdoms of life. We believe that our platform will be important as further roles for glycoRNAs in disease emerge, placing glycoRNAs in line with the misregulation of glycoproteins and glycolipids in human pathology [64]. Altogether, the AquIRE platform is a useful tool for the analysis of bulk RNA modifications with its flexibility and scalability providing the potential for discovery science across multiple aspects of RNA biology.

## Supplementary Material

gkag080_Supplemental_Files

## Data Availability

The data underlying this article are available in the article and in its online supplementary material.

## Notes

Current address: Lady Davis Institute, Department of Experimental Medicine, McGill University, Montreal, Quebec, H3T 1E2,Canada
