# Supplementary material for: AquIRE reveals the mechanisms of clinically induced RNA damage and the conservation and dynamics of glycoRNAs
Source: Nucleic Acids Res. 2026 Feb 5;54(4):gkag080. doi: 10.1093/nar/gkag080 (PMC12873605; doi:10.1093/nar/gkag080)
Supplement: gkag080_Supplemental_Files [file gkag080_supplemental_files.zip › Supplemental Figures.pdf]

Supplemental Figure 1

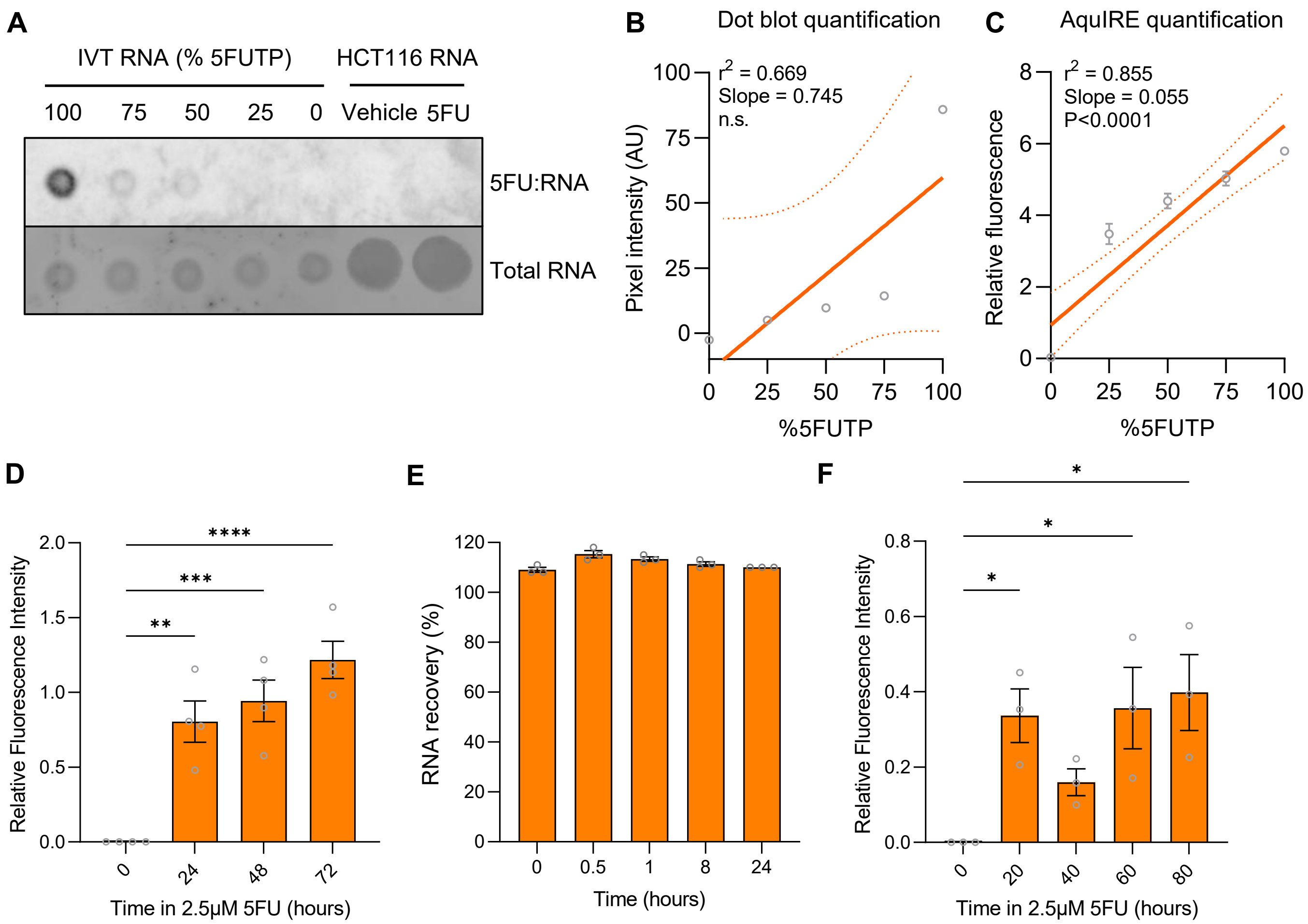

A) RNA from *in vitro* transcriptions (IVT) with the stated percentage of 5FUTP included in place of UTP or from HCT116 cells treated with 2.5μM 5FU for 24hours was analysed by RNA dot blot. 5FU:RNA was detected using anti-BrdU antibody with an HRP-conjugated secondary antibody. Total RNA was visualised using methylene blue. B) Pixel intensity was calculated for the IVT RNA and plotted against the known incorporation of 5FUTP. The orange line plots a simple linear regression, and the dashed line is the 95% confidence interval. Details of the linear regression fit are inset into the graph. Data are from one replicate. C) The same samples as in B were analysed by AquIRE and again expressed as a linear regression. Data are from at least two technical replicates (i.e. different AquIRE assays) of the same IVT RNA sample. D) Graph plots the mean relative fluorescence intensity for 5FU incorporation into RNA at the shown timepoints. These data are from 3 biological replicates, one of which is represented in Figure 1D. Significance was tested using an ANOVA with Šídák multiple comparison testing. E) RNA recovery was determined as the amount of RNA recovered as a percentage of RNA input, following the AquIRE assay shown in Figure 1E. Data are technical triplicates from one AquIRE assay. F) Data as in D but with one of the biological replicates depicted in Figure 1G. Significance compared for all timepoints relative to 0 hours was tested using an ANOVA with Šídák multiple comparison testing. * P<0.05, ** P<0.01, *** P<0.001, **** P<0.001

Supplemental Figure 2

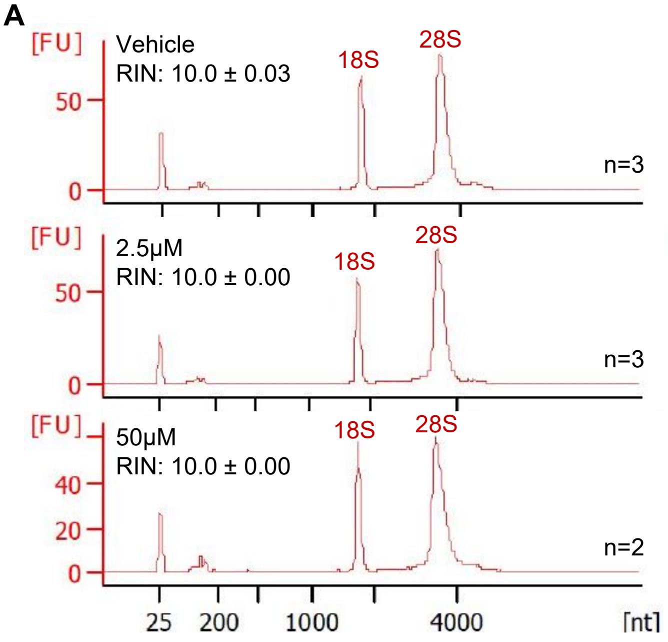

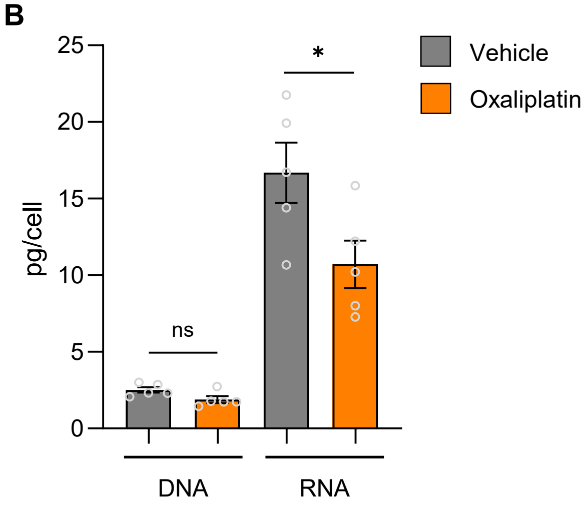

A) Representative TapeStation traces of RNA extracted from HCT116 cells following 24-hour treatment with the indicated does of oxaliplatin. The mean RIN score from the indicated number of biological replicates is inset into the traces ±SEM. B) DNA and RNA was extracted from 1 million vehicle or 2.5μM oxaliplatin treated HCT116 cells using Norgen's RNA/DNA Purification Kit. The quantity of DNA and RNA per cell was then calculated from the nucleic acid yield divided by cell number. The bars show the average of 5 biological replicates ±SEM. Significance for each nucleic acid was analysed by paired t test. * P<0.05

Supplemental Figure 3

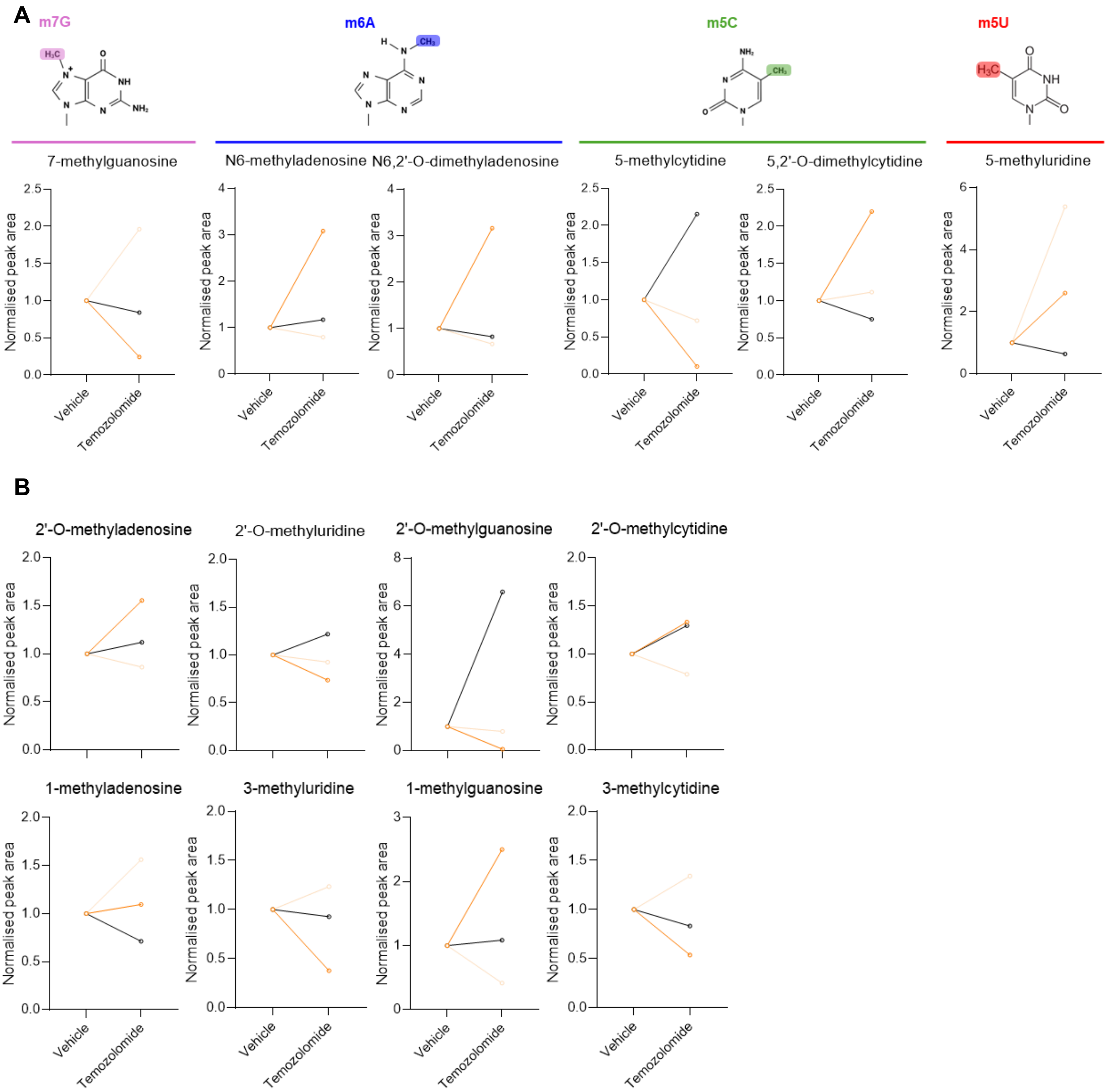

A) A172 glioblastoma cells were treated with vehicle or 2mM DMSO for 30mins, followed by total RNA extraction. Triplicate samples were analysed by LC-MS for select methylated nucleotides as depicted in schematics at the top of the panel. Normalised peak intensities were converted to fold changes and plotted for each replicate in a different colour. Each replicates across all panels in this figure retain the same colour. The samples were the same as those used in Figure 3C+D. B) From the same LC-MS analysis performed in (A), the fold changes in other methylated nucleotides are plotted. These methylations are known to stall reverse transcription due to 2'O backbone methylation (top row) or disrupting Watson Crick base pairing (bottom row).

Supplemental Figure 4

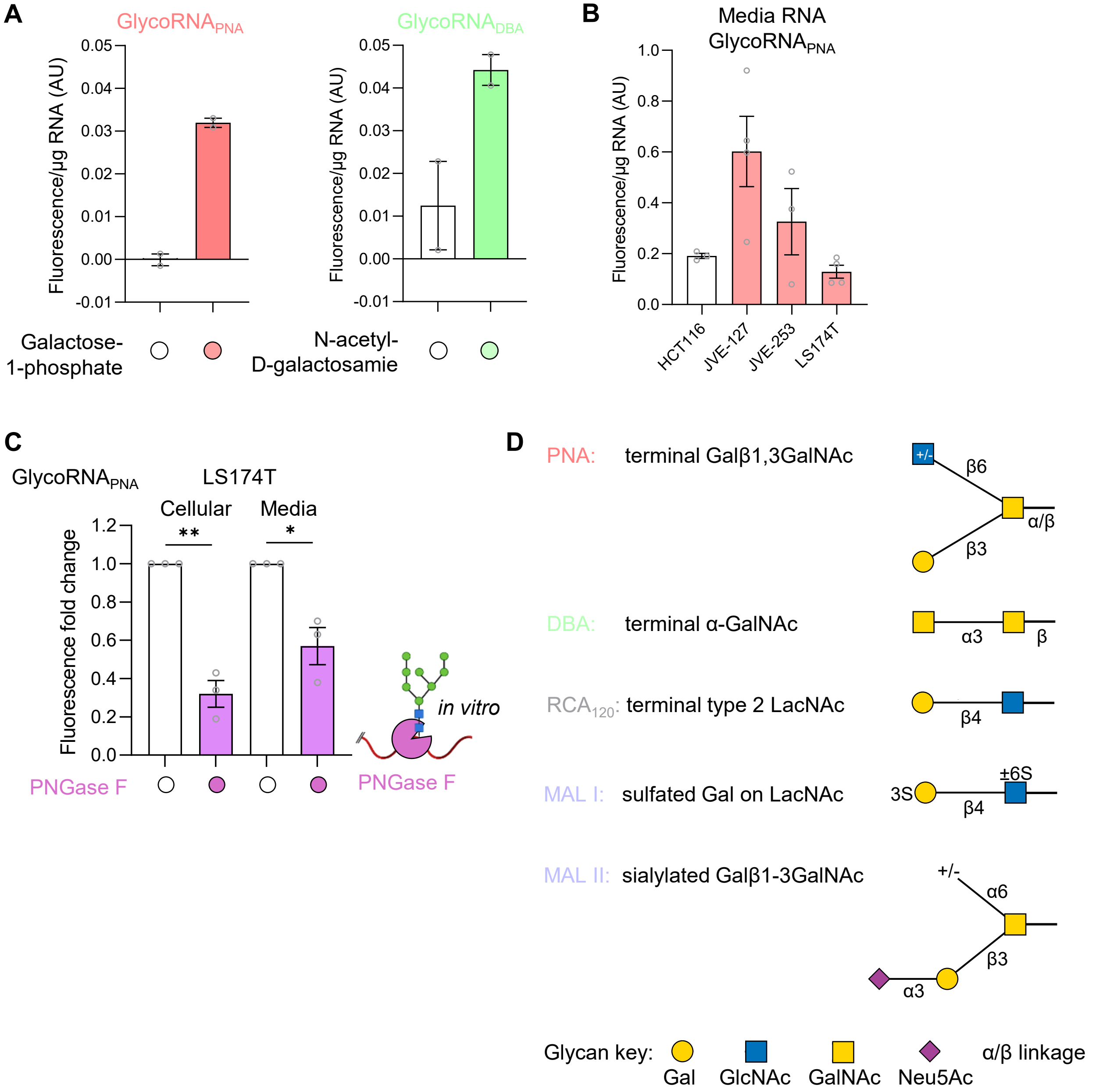

A) RNA extracted from LS174T cells was analysed for glycoRNA$_{PNA}$ or glycoRNA$_{DBA}$ and fluorescence signal eluted by incubation with 100mM of competitive soluble sugar – galactose-1-phosphate for PNA and N-acetyl-D-galactosamine for DBA. Data are presented for N=2 biological replicates ±SEM compared to incubation without sugars. B) RNA precipitated from the cell-free growth media of four colorectal cancer cell lines was analysed for glycoRNA$_{PNA}$ levels. Values are plotted as the fluorescence per µg of RNA from 3 biological replicates ±SEM after normalisation to IVT RNA. HCT116 cells (white bar) are non-mucinous in origin, while the other cells lines (red bars) are mucinous. C) RNA from LS174T cells or growth media was digested with PNGase F to remove N-glycans, followed by AquIRE detect glycoRNA$_{PNA}$. Graphs show the fold change in fluorescence after normalisation against IVT RNA for 3 biological replicates ±SEM. Significance was tested by paired t test. D) Representation of the binding specificities of the lectins used in this study drawn with GlycoGlyph. Lectin names are showed in coloured text, matching the colours in Figure 5C. * P<0.05, ** P<0.01

Supplemental Figure 5

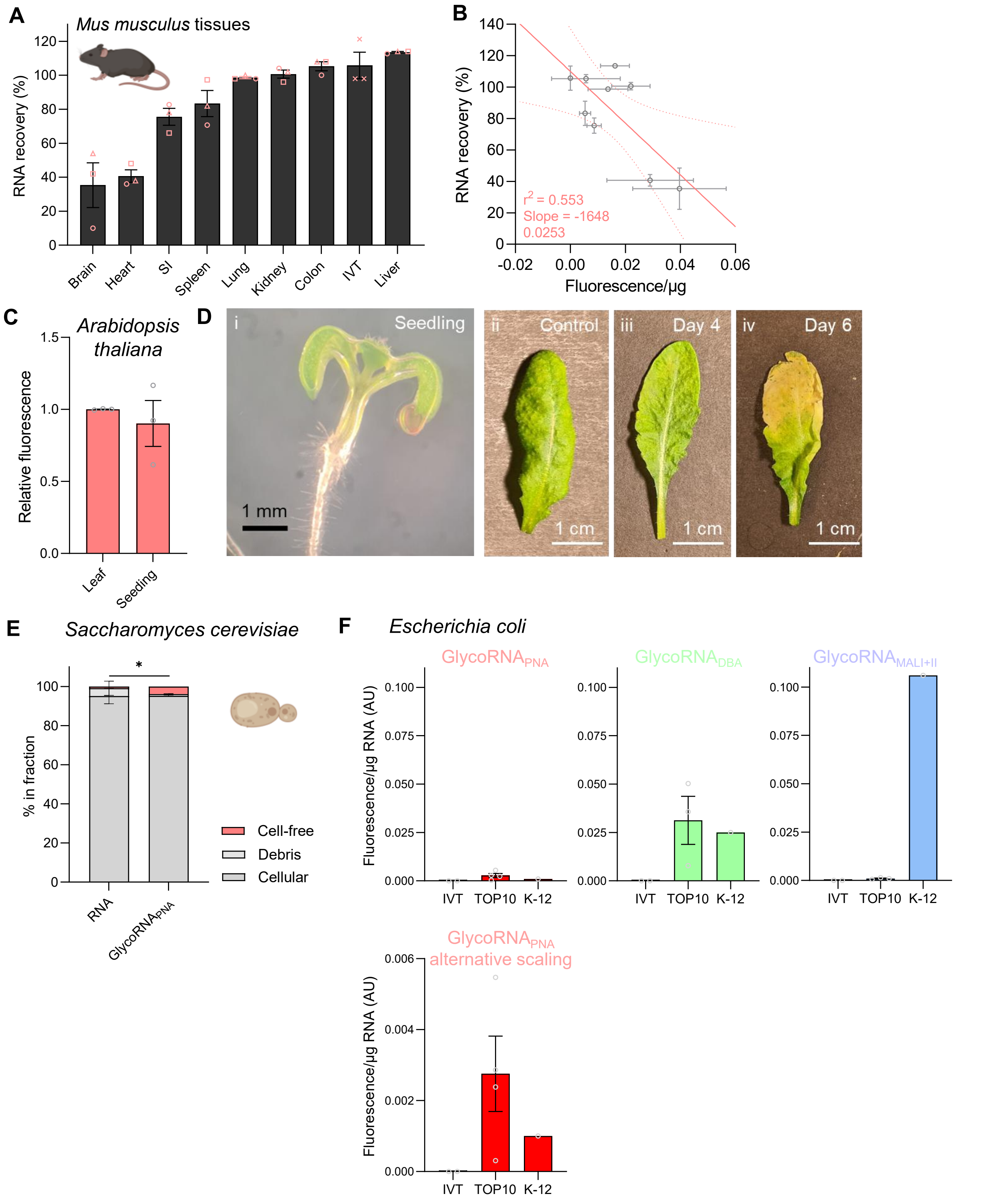

Supplemental Figure 5

A) The percentage of RNA recovered from AquIRE analyses from different mouse samples and an IVT RNA sample in Figure 6A are plotted. Each shape represents an individual animal of 3 biological replicates. B) The scatter graph plots the fluorescence data from Figure 6A against the percent recovery of RNA as shown in A. The red line shows a simple linear regression, and the dashed lines are the 95% confidence interval. Data of the fit of the linear regression are inset into the plot. C) RNA was extracted from *Arabidopsis* leaves (as analysed in Figure 6B) and glycoRNA$_{PNA}$ levels compared to RNA extracted from whole 7-day old seedlings. Data are plotted as the mean fold change in fluorescence from the leaf samples for 3 independent plants. D) Dark-induced senescence was induced in *Arabidopsis thaliana* Col-0 by wrapping selected leaves in aluminium foil while on the plant. Representative images of i) a 7-day old seedling ii) a leaf exposed to light, 8h per day, for 6 days. iii) a leaf after 4 days of the dark and iv) a leaf after 6 days of the dark. The leaves come from the same plant. E) W303-1A *Saccharomyces* were grown to OD0.8 then pelleted at low speed for cell RNA extraction. The supernatant was spun again to isolate cell debris, leaving a cell-free supernatant. RNA was extracted from all 3 fractions (cell pellet, cell debris and cell-free media) and analysed for glycoRNA$_{PNA}$ by AquIRE. Data are the fraction, as a percent, of the RNA and glycoRNA$_{PNA}$ signal found in each fraction from two biological replicates ±SEM. Significance was tested using a 2-way ANOVA with Šídák multiple comparison testing comparing the cell-free signal to the sum of the two other fractions. F) Total cellular RNA extracted from two independent strains of *Escherichia coli* were analysed for glycoRNA$_{PNA}$, glycoRNA$_{DBA}$, glycoRNA$_{MALI-II}$ by AquIRE. Data are presented as the fluorescence per µg or RNA in each case, following normalisation to the signal from IVT RNA. The top graphs retain the same y-axis scale for comparison between lectins, while the lower graph shows alternative scaling of the PNA plot. Data are the mean, expressed ±SEM. A single replicate was analysed for the commercially sourced K-12 strain RNA. * P<0.05

Supplemental Figure 6

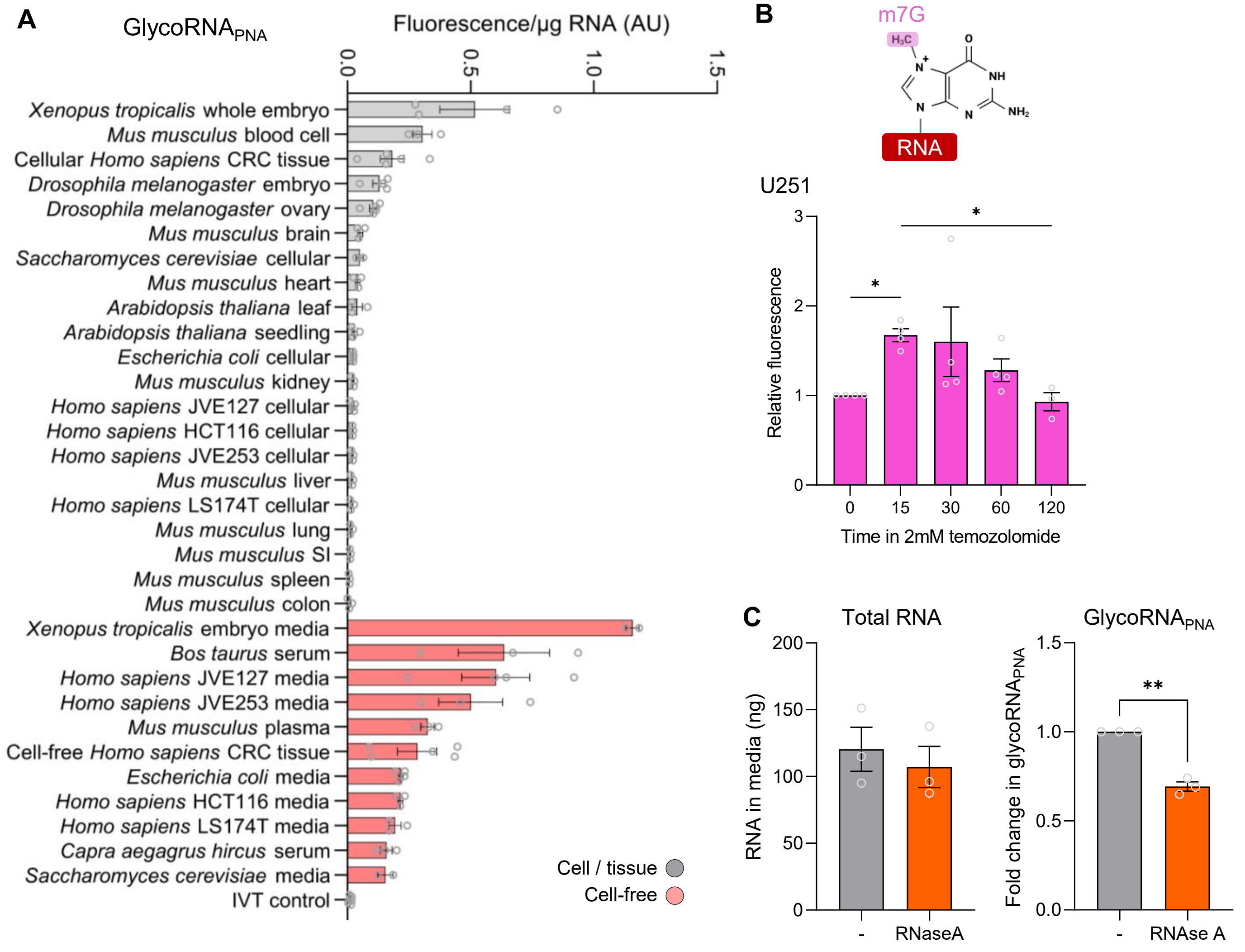

A) A single graph showing the relative levels of glycoRNA$_{PNA}$ across the biological samples analysed in this work. Data are plotted as the raw (not normalised to IVT) values for each sample, plus an IVT control in the final row. Data are from a minimum of 2 biological replicates, with individual replicate numbers visible from the small grey circles on the figure. Data are the mean values plotted ±SEM. Cell/tissue-based samples are plotted in rank order in grey. Cell-free samples are plotted in rank order below this in red. B) RNA was extracted from U251 glioblastoma cells following treatment with 2mM temozolomide for the indicated times. Left is a graph of the mean m7G signal detected by AquIRE from at least 3 biological replicates, plotted with the SEM. Significance was determined by mixed affects analysis. Right shows the methylation of guanosine at position 7 (pink) that is being detected. C) RNA was extracted from the media of HCT116 cells treated with 200µg/mL RNase A or no digestion for 72 hours. The total yield of RNA from extraction is plotted on the left graph and the glycoRNA$_{PNA}$ content detected by AquIRE on the right. Both graphs plot the mean of 3 biological replicates, plotted as grey circles, ±SEM. Significance was tested by paired t test. * P<0.05, ** P<0.01
