# Supplementary material for: AquIRE reveals the mechanisms of clinically induced RNA damage and the conservation and dynamics of glycoRNAs
Source: Nucleic Acids Res. 2026 Feb 5;54(4):gkag080. doi: 10.1093/nar/gkag080 (PMC12873605; doi:10.1093/nar/gkag080)
Supplement: gkag080_Supplemental_Files [file gkag080_supplemental_files.zip › Supplemental Table S1.docx]

***Supplemental Table S1. Reagents and resources used in this study***

| **Reagent or resource** | **Supplier** | **Identifier** |  |
| --- | --- | --- | --- |
| **Antibodies** | | | |
| Mouse monoclonal anti BrdU | Sigma Aldrich | Cat #B8434 |  |
| Rat monoclonal anti cisplatin modified DNA | Novus Biologicals | Cat #NBP2-50165 |  |
| Mouse anti 7-methylguanosine (m7G) | MBL Life Sciences | Cat #RN017M |  |
| Rabbit anti N6-methyladenosine (m6A) | EpigenTek / Insight Biotechnology | Cat #A-1802-020 |  |
| Mouse anti 5-methylcytosine (m5C) | EpigenTek / Insight Biotechnology | Cat #A-1014 |  |
| Mouse monoclonal anti pseudouridine | MBL Life Sciences | Cat #D347-3 |  |
| Biotin-XX conjugated goat polyclonal anti mouse IgG1 | Invitrogen | Cat #A10519 |  |
| Biotin-XX conjugated goat polyclonal anti rabbit IgG1 | Invitrogen | Cat #B2770 |  |
| Biotin-XX conjugated goat polyclonal anti rat IgG | Invitrogen | Cat #31830 |  |
| **Lectins** |  |  |  |
| Biotinylated Peanut Agglutinin (PNA) | Vector Laboratories | Cat #B-1075-5 |  |
| Biotinylated Dolichos Biflorus Agglutinin (DBA) | Vector Laboratories | Part of Cat # BK-1000 |  |
| Biotinylated Maackia Amurensis Lectin (MAL I+II) | CliniSciences | Cat #21511113-1 |  |
| Biotinylated Ricinus communis Agglutinin (RCA_120_) | Vector Laboratories | Part of Cat # BK-1000 |  |
| **Bacterial strains** |  |  |  |
| One Shot TOP10 Chemically Competent *E. coli* | Fisher Scientific/ Invitrogen | Cat # 10666493 |  |
| **Biological samples** |  |  |  |
| Human primary and metastatic colorectal cancer tissue | Manchester Cancer Research Centre Biobank | <https://www.mcrc.manchester.ac.uk/research/mcrc-biobank/about-the-mcrc-biobank/> |  |
| *E. Coli* total RNA from K-12 strain | Zyagen / amsbio | Cat #ECR-310 |  |
| **Chemicals, media and proteins** | | |  |
| Pregnant mare serum gonadotrophin | MSD Animal Health | Cat #369397 |  |
| Human chorionic gonadotrophin | MSD Animal Health | Cat# 377384 |  |
| MMR media | Homemade | N/A |  |
| Ionomycin | Sigma Aldrich | Cat # I3909 |  |
| Guanidine hydrochloride | Fisher Scientific | Cat #10543325 |  |
| Galactose-1-phosphate | Cambridge Bioscience | Cat# HY-113143A |  |
| N-acetyl-D-galactosamine | Sigma Aldrich | Cat# A2795 |  |
| Streptavidin-conjugated Alexa Fluor™ 488 | Thermo Fisher | Cat #S11223 |  |
| RNAlater^TM^ | Invitrogen | Cat #AM7020 |  |
| Trizol | Invitrogen | Cat #15596018 |  |
| Glycoblue | Thermo Fisher | Cat #AM9515 |  |
| RiboLock | Fisher Scientific | Cat #10389109 |  |
| 5-Fluorouracil (5FU) | Sigma Aldrich | Cat #F6627 |  |
| Oxaliplatin | Merck | Cat #PHR1528 |  |
| Cisplatin | Stratech Scientific | Cat #A8321-APE-100 |  |
| Carboplatin | Stratech Scientific | Cat #A2171-APE-100 |  |
| Temozolomide | Cayman Chemical | Cat #T2577 |  |
| STM2457 | Sigma Aldrich | Cat #34280 |  |
| NGI-1 | Sigma Aldrich | Cat # SML1620 |  |
| Benzyl-α-GalNAc | Sigma Aldrich | Cat #B4894 |  |
| PNGase F | New England Biolabs | Cat # P0704 |  |
| Random primers | Promega | Cat #C118A |  |
| dNTP mix | Invitrogen | Cat #18427013 |  |
| PseudoUTP | Stratech | Cat #NU-1139S-JEN |  |
| m6ATP | Enzo | Cat #JBS-NU-1101 |  |
| 5FUTP | Jena Bioscience | Cat #RNT-202 |  |
| 96 Well Black Plates | Life Technologies | Cat #237105 |  |
| Yeast Nitrogen Base w/o amino acid | Difco Laboratories | Cat #291920 |  |
| Kaiser Complete SC mixture | Formedium | Cat #DSCK1000 |  |
| D-glucose | Fisher Scientific | Cat #G/0500/61 |  |
| Murashige and Skoog basal medium | Duchefa | Cat #M0221 |  |
| DMEM | Sigma Aldrich | Cat #D6546 |  |
| RPMI | Sigma Aldrich | Cat #R0883 |  |
| MEM | Gibco | Cat #11514426 |  |
| Advanced DMEM / F12 | Fisher Scientific | Cat #11540446 |  |
| HEPES | Fisher Scientific | Cat # 11560496 |  |
| L-glutamine | Sigma Aldrich | Cat #G7513 |  |
| GlutaMAX^TM^ | Thermo Scientific | Cat #35050038 |  |
| Foetal Bovine Serum | Sigma Aldrich | Cat #F7524 |  |
| Tet-approved Foetal Bovine Serum | Thermo Scientific | Cat #A47361-01 |  |
| Normal Goat Serum | Sigma Aldrich | Cat #G9023 |  |
| Normal Goat Serum | Vector Laboratories | Cat #S1000-20 |  |
| Normal Goat Serum | Thermo Scientific | Cat #01-6201 |  |
| Non-essential amino acids | Thermo Scientific | Cat #11140035 |  |
| Penicillin Streptomycin (PenStrep) | Sigma Aldrich | Cat #P0781 |  |
| *E. Coli* Poly(A) polymerase | New England Biolabs | Cat #M0276 |  |
| Collagenase II | Merck | Cat #C2-28 |  |
| Sterile EDTA | VWR International | Cat #E177 |  |
| Collagenase inhibitor | Merck | Cat #234140 |  |
| Poly-L-lysine | Merck | Cat #P8920 |  |
| DAPI | Merck | Cat #D9542 |  |
| Cycloheximide | Merck | Cat #01810 |  |
| Monarch RNase A | New England Biolabs | Cat #T3018 |  |
| **Commercial assays** |  |  |  |
| Vivaspin 20, 5kDa cut off | Sartorius | Cat # 10325271 |  |
| Disposable pestle | Fisher Scientific | Cat #13236679 |  |
| Heparin tubes | Teklab | Cat #NH200PP |  |
| gentleMACS C tubes | Miltenyi Biotec | Cat #130-093-237 |  |
| gentleMACS M tubes | Miltenyi Biotec | Cat #130-094-392 |  |
| 100µm cell strainer | Starlab | Cat #CC8111-0102 |  |
| Sera-mag oligo(dT) coated beads | Cytivia | Cat #38152103010150 |  |
| Quick-RNA^TM^ Miniprep Kit | Zymo Research | Cat #R1055 |  |
| RNA Clean & Concentrator-5 | Zymo Research | Cat #R1013 |  |
| RNA/DNA Purification Kit | Norgen | Cat #48700 |  |
| Aurum Total RNA Mini Kit | BioRad | Cat #7326820 |  |
| SuperScript^TM^ II Reverse Transcriptase kit | Invitrogen | Cat #18064-014 |  |
| PowerTrack^TM^ SYBR^TM^ Green Master Mix | Applied Biosystems^TM^ | Cat #A46109 |  |
| HighYield T7 RNA Synthesis Kit | Jena Bioscience | Cat #RNT-202 |  |
| Streptavidin/biotin blocking kit | Vector Laboratories | Cat #SP2002 |  |
| Monarch Spin RNA Cleanup Kit | New England Biolabs | Cat #T2040 |  |
| Agilent Bioanalyzer | Agilent | N/A |  |
| Varioscan Lux Plate Reader | Thermo Scientific | N/A |  |
| UA6 UV Detector with fractionation system | Brandel | Cat #BR-188-5 |  |
| Foxy R1 Fraction Collector | Teledyne | N/A |  |
| Confocal spinning disc microscope | Olympus | N/A |  |
| QuantStudio 5-C Real-Time PCR System | Thermo Scientific | N/A |  |
| **Experimental models: Cell lines** |  |  |  |
| HCT116 | Gift from Stephen Taylor, University of Manchester | N/A |  |
| JVE-127 | DSMZ | Cat #ACC 813 |  |
| JVE-253 | DSMZ | Cat #ACC 823 |  |
| LS174T | European Collection of Cell Culture | Cat #87060401 |  |
| RKO | Gift from Stephen Taylor, University of Manchester | N/A |  |
| DLD-1 | Gift from Stephen Taylor, University of Manchester | N/A |  |
| A172 | Gift from Petra Hamerlik, University of Manchester | N/A |  |
| U251 | Gift from Petra Hamerlik, University of Manchester | N/A |  |
| **Experimental models: Organisms/strains** | | | |
| *Saccharomyces cerevisiae* W303-1A | ATCC | Cat #208352 |  |
| *Mus musculus* mixed background | CRUK Manchester Institute | N/A |  |
| *Xenopus tropicalis* | <https://xenopusresource.org/> | N/A |  |
| *Arabidopsis thaliana* ecotype Columbia (Col-0) | NASC, Nottingham UK | Cat #N1093 |  |
| *Drosophila melanogaster* (*y*^1^ *w*^67c23^) | Bloomington Drosophila Stock Centre | Cat # 6599 |  |
| **Oligonucleotides** |  |  |  |
| 18S rRNA forward primer:  AATTCCCAGTAAGTGCGGGT | This paper | N/A |  |
| 18S rRNA reverse primer:  CCTTCCGCAGGTTCACCTA | This paper | N/A |  |
| *GAPDH* forward primer:  GGAGCGAGATCCCTCCAAAAT | This paper | N/A |  |
| *GAPDH* reverse primer:  GGCTGTTGTCATACTTCTCATGG | This paper | N/A |  |
| *ACTB* forward primer:  CATGTACGTTGCTATCCAGGC | This paper | N/A |  |
| *ACTB* reverse primer:  CTCCTTAATGTCACGCACGAT | This paper | N/A |  |
| **Recombinant DNA** |  |  |  |
| pUC57-Curlcake 3 IVT template | Addgene; (25) | Cat #139342 http://n2t.net/addgene:139342 ; RRID:Addgene_139342 |  |
| **Software and algorithms** | | | |
| Thermo Scientific^TM^ Design and Analysis Software 2 | Thermo Scientific | Cat #16494480 |  |
| GraphPad Prism 10.0 | GraphPad Prism | https://www.graphpad.com/features |  |
| Image J | (26) | https://imagej.nih.gov/ij/ |  |
| SkanIt™ Software | Thermo Scientific | Cat #5187139 |  |
| PeakChart 3 | Brandel | Cat #2115388 |  |
