# Supplementary material for: AquIRE reveals the mechanisms of clinically induced RNA damage and the conservation and dynamics of glycoRNAs
Source: Nucleic Acids Res. 2026 Feb 5;54(4):gkag080. doi: 10.1093/nar/gkag080 (PMC12873605; doi:10.1093/nar/gkag080)
Supplement: gkag080_Supplemental_Files [file gkag080_supplemental_files.zip › Supplemental Table S2.docx]

***Supplemental Table S2: Selected patient and tumour details***

| **Tumour ID** | **MCRC-Om-001** | **MCRC-PN-003** | **MCRC-Ca-005** | **MCRC-LM-007** | **MCRC-OM-008** |
| --- | --- | --- | --- | --- | --- |
| **Primary tumour** | Caecum | Transverse colon | Caecum | Caecum | Caecum |
| **Metastatic site** | Omentum | Left pararenal nodule | N/A | Liver | Omentum |
| **Site sampled** | Metastasis | Metastasis | Primary | Metastasis | Metastasis |
| **Date sampled** | 23/05/2024 | 18/07/2024 | 22/08/2024 | 11/10/2024 | 06/11/2024 |
| **Sample weight** | 4000mg | 1800mg | 1100mg | 1000mg | 1800mg |
| **Age (years)** | 66 | 60 | 52 | 56 | 81 |
| **Mucinous** | Yes | Yes | Yes | No | Yes |
| **MMR status** | MSS | Unknown | MSS | MSS | MSS |
| ***KRAS*** | WT | WT | WT | Gly12Val | WT |

Details of the patients and selected tumour characteristics for the samples used in this study to identify cellular and cell-free glycoRNA expression.
