# Supplementary material for: AquIRE reveals the mechanisms of clinically induced RNA damage and the conservation and dynamics of glycoRNAs
Source: Nucleic Acids Res. 2026 Feb 5;54(4):gkag080. doi: 10.1093/nar/gkag080 (PMC12873605; doi:10.1093/nar/gkag080)
Supplement: gkag080_Supplemental_Files [file gkag080_supplemental_files.zip › Supplemental Table S3.docx]

***Supplemental Table S3: Normalized peak from LC-MS of canonical and modified nucleosides***

| **Nucleoside** | **Symbol** | **Normalized Peak Area** | | | | | | **Fold Change** | | | | **P-value** |  |  |
| --- | --- | --- | --- | --- | --- | --- | --- | --- | --- | --- | --- | --- | --- | --- |
|  |  | **Vehicle 1** | **TMZ 1** | **Vehicle 2** | **TMZ 2** | **Vehicle 3** | **TMZ 3** | **Rep 1** | **Rep 2** | **Rep 3** | **Average** |  |  |  |
| cytidine | C | 81849 | 55011 | 267472 | 242246 | 79517 | 105378 | 0.67 | 0.91 | 1.33 | 0.94 | 0.66 |  | Canonical nucleotides |
| guanosine | G | 42578 | 38539 | 34372 | 34809 | 18498 | 31735 | 0.91 | 1.01 | 1.72 | 1.10 | 0.60 |  |  |
| adenosine | A | 26475 | 28685 | 24622 | 24025 | 12590 | 23399 | 1.08 | 0.98 | 1.86 | 1.20 | 0.35 |  |  |
| uridine | U | 5325 | 3105 | 16064 | 15689 | 6528 | 7653 | 0.58 | 0.98 | 1.17 | 0.95 | 0.66 |  |  |
| 7-methylguanosine | m7G | 50 | 42 | 28 | 55 | 33 | 8 | 0.84 | 1.96 | 0.24 | 0.95 | 0.91 |  | Methylated nucleotides analysed by AquIRE, plus 5-methyluridine |
| N6-methyladenosine | m6A | 1111 | 1301 | 1387 | 1104 | 485 | 1495 | 1.17 | 0.80 | 3.08 | 1.31 | 0.50 |  |  |
| N6,2'-O-dimethyladenosine | m6Am | 292 | 241 | 124 | 83 | 86 | 272 | 0.83 | 0.67 | 3.16 | 1.19 | 0.72 |  |  |
| 5-methylcytidine | m5C | 71 | 153 | 317 | 228 | 193 | 20 | 2.15 | 0.72 | 0.10 | 0.69 | 0.51 |  |  |
| 5,2'-O-dimethylcytidine | m5Cm | 44 | 33 | 43 | 48 | 35 | 77 | 0.75 | 1.12 | 2.20 | 1.30 | 0.52 |  |  |
| 5-methyluridine | m5U | 88 | 56 | 26 | 140 | 10 | 26 | 0.64 | 5.38 | 2.60 | 1.79 | 0.53 |  |  |
| 2'-O-methyluridine | Um | 15351 | 18727 | 21585 | 19997 | 19249 | 14189 | 1.22 | 0.93 | 0.74 | 0.94 | 0.70 |  | Methylated nucleotides known to impede reverse transcription: 2'-O-methylation or Watson-Crick face methylations |
| 2'-O-methyladenosine | Am | 4738 | 5314 | 5500 | 4742 | 3112 | 4843 | 1.12 | 0.86 | 1.56 | 1.12 | 0.55 |  |  |
| 2'-O-methylguanosine | Gm | 5 | 33 | 31 | 25 | 81 | 5 | 6.60 | 0.81 | 0.06 | 0.54 | 0.62 |  |  |
| 2'-O-methylcytidine | Cm | 338 | 438 | 925 | 730 | 504 | 672 | 1.30 | 0.79 | 1.33 | 1.04 | 0.85 |  |  |
| 1-methyladenosine | m1A | 1556 | 1109 | 1314 | 2054 | 619 | 678 | 0.71 | 1.56 | 1.10 | 1.10 | 0.77 |  |  |
| 1-methylguanosine | m1G | 46 | 50 | 84 | 35 | 6 | 15 | 1.09 | 0.42 | 2.50 | 0.74 | 0.58 |  |  |
| 3-methylcytidine | m3C | 11319 | 9427 | 7453 | 9998 | 11729 | 6308 | 0.83 | 1.34 | 0.54 | 0.84 | 0.56 |  |  |
| 3-methyluridine | m3U | 499 | 463 | 308 | 380 | 779 | 294 | 0.93 | 1.23 | 0.38 | 0.72 | 0.47 |  |  |
